# Mcl-1 mediates intrinsic resistance to RAF inhibitors in mutant BRAF papillary thyroid carcinoma

**DOI:** 10.1038/s41420-024-01945-0

**Published:** 2024-04-15

**Authors:** Maria R. Cavallo, Jacob C. Yo, Kayla C. Gallant, Camille J. Cunanan, Amirali Amirfallah, Marzieh Daniali, Alyssa B. Sanders, Andrew E. Aplin, Edmund A. Pribitkin, Edward J. Hartsough

**Affiliations:** 1 Department of Pharmacology and Physiology, Drexel University College of Medicine, Philadelphia, PA 19102 USA; 2 Graduate School of Biomedical Sciences and Professional Studies, Drexel University College of Medicine, Philadelphia, PA 19102 USA; 3 Sidney Kimmel Cancer Center, Philadelphia, PA 19107 USA; 4 Departments of Pharmacology, Physiology and Cancer Biology, Thomas Jefferson University, Philadelphia, PA 19107 USA; 5 Departments of Otolargynology-Head & Neck Surgery, Thomas Jefferson University, Philadelphia, PA 19107 USA

**Keywords:** Cancer therapeutic resistance, Targeted therapies

## Abstract

Papillary thyroid carcinoma (PTC) is the most frequent form of thyroid cancer. PTC commonly presents with mutations of the serine/threonine kinase BRAF (BRAF^V600E^), which drive ERK1/2 pathway activation to support growth and suppress apoptosis. PTC patients often undergo surgical resection; however, since the average age of PTC patients is under 50, adverse effects associated with prolonged maintenance therapy following total thyroidectomy are a concern. The development of mutant-selective BRAF inhibitors (BRAFi), like vemurafenib, has been efficacious in patients with metastatic melanoma, but the response rate is low for mutant BRAF PTC patients. Here, we assay the therapeutic response of BRAFi in a panel of human PTC cell lines and freshly biopsied patient samples. We observed heterogeneous responses to BRAFi, and multi-omic comparisons between susceptible and resistant mutant BRAF PTC revealed overrepresented stress response pathways and the absence of compensatory RTK activation – features that may underpin innate resistance. Importantly, resistant cell lines and patient samples had increased hallmarks of failed apoptosis; a cellular state defined by sublethal caspase activation and DNA damage. Further analysis suggests that the failed apoptotic phenotypes may have features of “minority mitochondrial outer membrane permeabilization (MOMP)” – a stress-related response characterized by fragmented and porous mitochondria known to contribute to cancer aggressiveness. We found that cells presenting with minority MOMP-like phenotypes are dependent on the apoptotic regulator, Mcl-1, as treatment with the Mcl-1 inhibitor, AZD5991, potently induced cell death in resistant cells. Furthermore, PI3K/AKT inhibitors sensitized resistant cells to BRAFi; an effect that was at least in part associated with reduced Mcl-1 levels. Together, these data implicate minority MOMP as a mechanism associated with intrinsic drug resistance and underscore the benefits of targeting Mcl-1 in mutant BRAF PTC.

## Introduction

Mutations in the RAS-RAF-MEK-ERK pathway are major drivers of human malignancy; the serine/threonine kinase BRAF is mutated in up to 8% of all human cancers [1]. The most common mutation occurs at position 600, where valine is substituted to a glutamic acid (V600E), mimicking the phospho-activation of neighboring residues. BRAF^V600E^ is the driver oncogene in ~50% of thyroid carcinomas [2]. Of these, papillary thyroid carcinoma (PTC) is the most prevalent subtype, representing ~90% of total thyroid carcinomas [3]. Surgical intervention is often successful for PTC patients; however, total thyroidectomy necessitates lifelong hormone replacement therapy (HRT) [3]. Due to the low median age of PTC diagnosis [4], patients are often treated with HRT for decades and treatment is associated with adverse effects, like secondary cancers [5] and reduced bone density [6].

While total thyroidectomy is the recommended first-line therapy for some PTC patients with more advanced disease, targeted therapies designed to eliminate cancerous lesions selectively and spare non-diseased tissue would be clinically beneficial. FDA-approved BRAF^V600E^ targeted inhibitors (BRAFi) like vemurafenib and dabrafenib have been explored for those with unresectable or radioactive iodine (RAI) resistant thyroid carcinomas [7–9]. However, the response rate is limited, and resistance is inevitable [10–12].

BRAFi resistance is a common clinical hurdle for BRAF^V600E^ metastatic melanoma patients. In many cases, drug treatment effectively shrinks tumors; however, a subset of tumor cells employ adaptive resistance – cellular programming that quickly responds to ERK1/2 inhibition to promote survival [13, 14]. This process likely leads to sustained minimal residual disease, facilitating eventual relapse [15–17]. Parallel to this, some tumor cells possess intrinsic or innate resistance to chemotherapy [18], targeted agents [19, 20], and immunotherapy [21]. Notably, compared to melanomas, mutant BRAF thyroid cancers are more refractory to BRAFi treatment as evidenced by a lower overall response rate [10, 22] – likely indicating an increased capacity for innate BRAFi resistance.

The efficacy of BRAFi therapy is reliant on the inhibition of ERK1/2 signaling and subsequent activation of apoptotic-like programming leading to cell death. Permeabilization of the mitochondrial membrane, Mitochondrial Outer Membrane Permeabilization (MOMP), is a critical component of the apoptotic process as it dysregulates energetics and amplifies intracellular stresses like caspase activation [23]. Recent studies have identified a mechanism, “minority MOMP”, that exhibits incomplete mitochondrial permeabilization and is associated with sublethal caspase activation [24]. Minority MOMP and parallel phenomena of “failed apoptosis” are known to promote oncogenesis and cancer aggressiveness through low levels of caspase activation, genomic instability, and activation of proliferative/invasive signaling [25, 26]. Importantly, sublethal apoptosis is linked to the expression of pro-survival Bcl-2 proteins colocalized with fragmented mitochondria [27], and minority MOMP can protect BRAF^V600E^ melanoma cells from targeted therapies [25].

In this study, we assayed a panel of human PTC cell lines and patient samples for BRAFi susceptibility. Paralleling clinical observations, we found heterogeneous responses that indicate innate resistance. As there was no obvious genetic rationale for these findings, we employed multi-omic approaches to better understand the varied BRAFi effects. To this end, we observed phenotypes consistent with minority MOMP including elevated inflammatory signaling, DNA damage, and mitochondrial fragmentation in intrinsic BRAFi resistant PTC. We found that the anti-apoptotic Bcl-2 family member, Mcl-1, was associated with maintaining the balance of minority MOMP in innately resistant cells, as Mcl-1 inhibition potently induced cell death. Furthermore, BRAFi in combination with PI3K/AKT inhibitors could overcome Mcl-1 mediated minority MOMP resistance. Together, our data illustrate a new mechanism of innate resistance in mutant BRAF PTC and highlight Mcl-1 as an important regulator of BRAFi susceptibility.

## Results

### Mutant BRAF PTC cell lines have heterogeneous responses to BRAF inhibitors

To better understand how BRAFi affects PTC, we obtained a panel of human cell lines (MDA-T32, -T41, -T68, -T85) [28]. The mutational status of each cell line was previously characterized: MDA-T32 and MDA-T41 are both BRAF^V600E^ mutant PTC cell lines. MDA-T68 has NRAS^Q61K^ mutation and is wildtype for BRAF. Interestingly, MDA-T85 has activating mutations in both BRAF and HRAS [28]. This cell panel was treated with increasing concentrations of the BRAFi PLX4720 (a vemurafenib analog), and PLX8394 (a next-generation “paradox breaker” BRAFi [29] known to overcome some forms of BRAFi resistance [29–31]). Trametinib was used as a positive control for pathway inhibition as MEKi has strong pre-clinical efficacy [32–34]. However, it is noteworthy that MEKi produces disappointing results as a monotherapy for PTC patients in clinical trials [35]. Western blot analysis showed that trametinib consistently reduced ERK1/2 activity, while both PLX4720 and PLX8394 had modest and heterogeneous effects (Supplemental Fig. 1). Notably, PLX4720 and not PLX8394 elicited a robust dose-dependent paradoxical ERK1/2 activation in the BRAF wildtype MDA-T68 cell line (Supplemental Fig. 1).

**Fig. 1:**
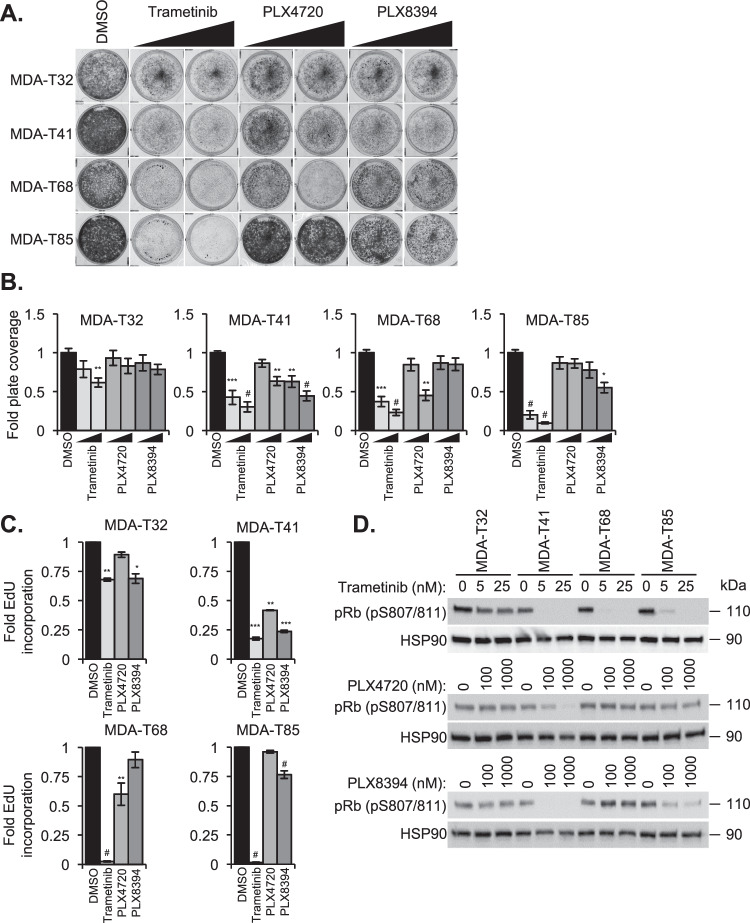
PTC cell lines have a differential response to BRAFi. **A** Representative crystal violet staining of PTC cells after a 6-day incubation in DMSO or increasing concentrations of trametinib (5, 25 nM), PLX4720 (100, 1000 nM), or PLX8394 (100, 1000 nM). **B** Quantification of crystal violet staining represented by fold plate coverage compared to DMSO treatment. **C** S-phase entry analysis via EdU incorporation of PTC cell lines treated with DMSO, trametinib (25 nM), PLX4720 (1000 nM), or PLX8394 (1000 nM). **D** Western blot analysis of Rb phosphorylation in PTC cell lines treated for 24 h with trametinib (top), PLX4720 (middle), and PLX8394 (bottom). Data points are representative of composite fold changes of at least three independent experiments with error bars signifying ±SEM (**B**, **C**). The * is indicative of *p* < 0.05, ** of *p* < 0.01, *** of *p* < 0.001, and # *p* < 0.0001 as determined by one-way ANOVA analysis with multiple comparisons (**B**, **C**).

We next assayed the effect of these drugs on proliferative potential. Crystal violet-based analysis paralleled results from ERK1/2 signaling. Trametinib largely blocked the growth of all cell lines tested, while PLX4720 and PLX8394 treatment had heterogeneous effects in the PTC cell lines (Fig. 1A, B). We found that MDA-T32 had a modest response to trametinib while resistant to PLX4720 and PLX8394. In contrast, the growth of MDA-T41 was suppressed by all three drugs. Furthermore, while PLX8394 had little effect on MDA-T68, PLX4720 treatment suppressed its proliferation (Fig. 1A, B), a result that may be linked to untenably high paradoxical ERK1/2 activation (Supplemental Fig. 1). These data were bolstered with EdU incorporation/S-phase entry assays to serve as a proxy for cell cycle progression. We found that MDA-T32 had a modest response to trametinib and PLX8394, while all three drugs potently blocked the cell cycle entry of MDA-T41. MDA-T68 and -T85 were both strongly affected by trametinib with heterogeneous responses to PLX4720 and PLX8394 (Fig. 1C). To further validate how these drugs affect the cell cycle, cell lines were dosed with inhibitors, and Rb phosphorylation was assayed via western blot. Mirroring EdU results, MDA-T32 was resistant to treatment, while MDA-T41 was largely susceptible (Fig. 1D). Additionally, treatment with PLX4720 and PLX8394 were associated with varied responses to Rb phosphorylation in MDA-T68 and -T85 (Fig. 1D).

**Fig. 2:**
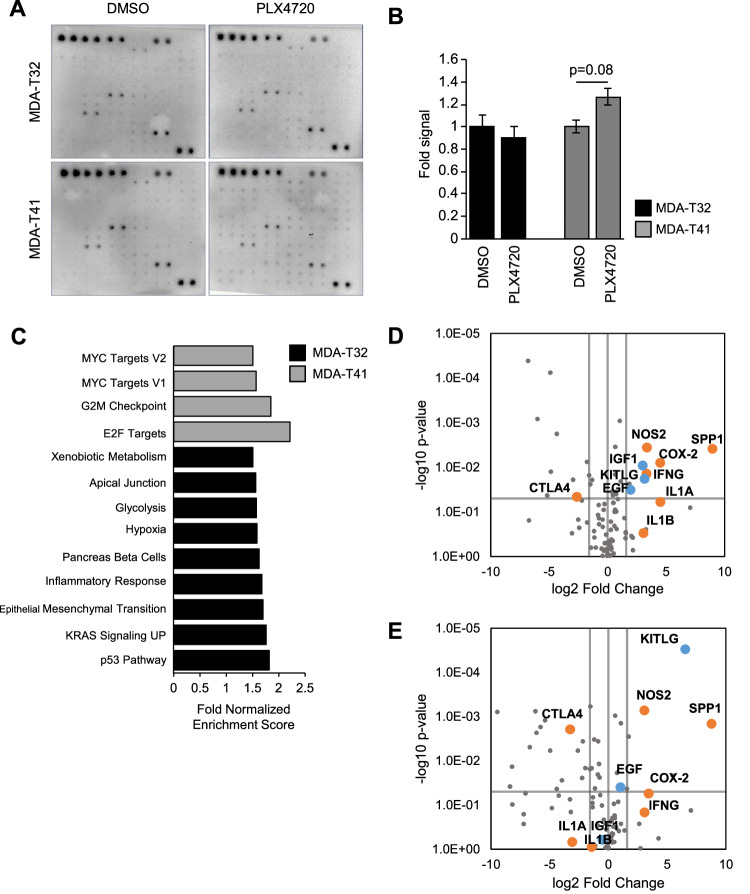
Resistant MDA-T32 PTC cell line has reduced adaptive response and higher inflammatory signaling. **A** PTC cell lines treated overnight with PLX4720 (1000 nM) were lysed and analyzed for RTK phosphorylation using the RayBio® C-Series Human Receptor Tyrosine Kinase Phosphorylation Antibody Array. **B** Composite intensity of staining across all 71 antibody targets in RayBio phosphorylation array to assess RTK signaling in MDA-T32 and MDA-T41 cells after 24-h incubation with DMSO or PLX4720 (1000 nM) were normalized to the intrinsic positive controls and graphed as a fold signal intensity. Error bars are ±SEM, *n* = 2. (RTK array antibody legend available in supplemental Fig. 2A). **C** Hallmark pathways enriched in MDA-T32 (black) and MDA-T41 (gray) bars. Data was obtained from [32] for use with Gene Set Enrichment Analysis (GSEA) [41] application for comparative analysis. Pathways with NES > 1.5, FDR < 0.05 are shown. **D**, **E** Volcano plot of Cancer Inflammatory and Immunity Crosstalk RT^2^ qPCR array comparing gene expression in MDA-T32 cells to that of MDA-T41 after 24-h treatment with DMSO (**D**) or PLX4720 (1000 nM) (**E**). Orange dots denote select differentially expressed genes in inflammatory signaling and blue dots, mitogenic signaling.

Since MDA-T32 and MDA-T41 have similar mutational status [28], yet vary in response to BRAFi (Fig. 1), we questioned if MDA-T32 possessed a form of intrinsic or innate resistance to BRAFi. We first assayed how BRAFi treatment stimulated receptor tyrosine kinase (RTK) activation. Enhanced RTK signaling is linked to adaptive and acquired resistance to targeted inhibitors in melanoma [13, 36, 37], colorectal [38, 39], and thyroid [12] malignancies. We utilized activated RTK antibody arrays to identify candidates that could be providing signaling associated with drug resistance. We found that BRAFi had no noticeable effects on MDA-T32, while MDA-T41 broadly upregulated RTK activation (Fig. 2A, B, Supplemental Fig. 2A), indicating innate resistance and a conventional adaptive response to reduced ERK1/2 pathway flux, respectively.

To better understand the potential mechanism underpinning the innate resistance phenotype in MDA-T32, we explored transcriptomic profiles. Published next-generation sequencing data of the broadly resistant MDA-T32 and susceptible MDA-T41 [40] were used for Gene Set Enrichment Analysis (GSEA) [41] to identify overrepresented pathways that may give insights into drug resistance. Overall, MDA-T32 had an enrichment of pathways associated with stress responses including: p53 Pathway, Inflammatory Response, and Hypoxia, while MDA-T41 was enriched for Cell Cycle and Mitogenic Pathways (Fig. 2C).

We next indexed the prevalence of the top 1% of differentially expressed genes as indicated by GSEA’s ranked gene list. Vascular adhesion molecule 1, VCAM1, is one of 3 genes (CCND2, SPP1/OPN, and VCAM1) that contributes to the enrichment score of more than one pathway (Supplemental Fig. 3A). Furthermore, VCAM1 expression is associated with more aggressive PTC [42] and has recently been implicated in therapy resistance of mutant BRAF PTC [43]. We questioned if VCAM1 expression is contributing to the refractory nature of MDA-T32. We probed our cell line panel and found that only MDA-T32 expressed VCAM1 and levels increased when cells were treated with PLX4720 (Supplemental Fig. 3B). Using siRNAs, we knocked down VCAM1 in MDA-T32 (Supplemental Fig. 3C) but did not observe increased susceptibility to inhibitors in crystal violet growth assays (Supplemental Fig. 3D) or S-phase entry (Supplemental Fig. 3E). Furthermore, we attempted to provide resistance to MDA-T41 cells via exogenous VCAM1 expression under doxycycline control (Supplemental Fig. 3F, G). However, expression of VCAM1 did not stabilize Rb phosphorylation (Supplemental Fig. 3F) or increase S-phase entry (Supplemental Fig. 3G), suggesting that in the cell lines we tested, VCAM1 alone is not responsible for targeted therapy resistance.

Since we did not observe an adaptive response to acute BRAFi treatment (Fig. 2A, B) and stress pathways were elevated in MDA-T32 (Fig. 2C), we used targeted qPCR arrays to obtain more direct insights into inflammation-associated gene expression differences between MDA-T32 and -T41. Interestingly, we found higher levels of the inflammatory genes interferon gamma (IFNG), nitric oxide synthase 2 (NOS2), prostaglandin-endoperoxide synthase 2 (PTGS2/COX2), and Osteopontin (SPP1/OPN) in MDA-T32 (Fig. 2D); of these, elevated expression of NOS2 and SPP1/OPN persisted with BRAFi treatment (Fig. 2E).

### Resistant MDA-T32 cells exhibit failed apoptosis and altered mitochondrial morphology

**Fig. 3:**
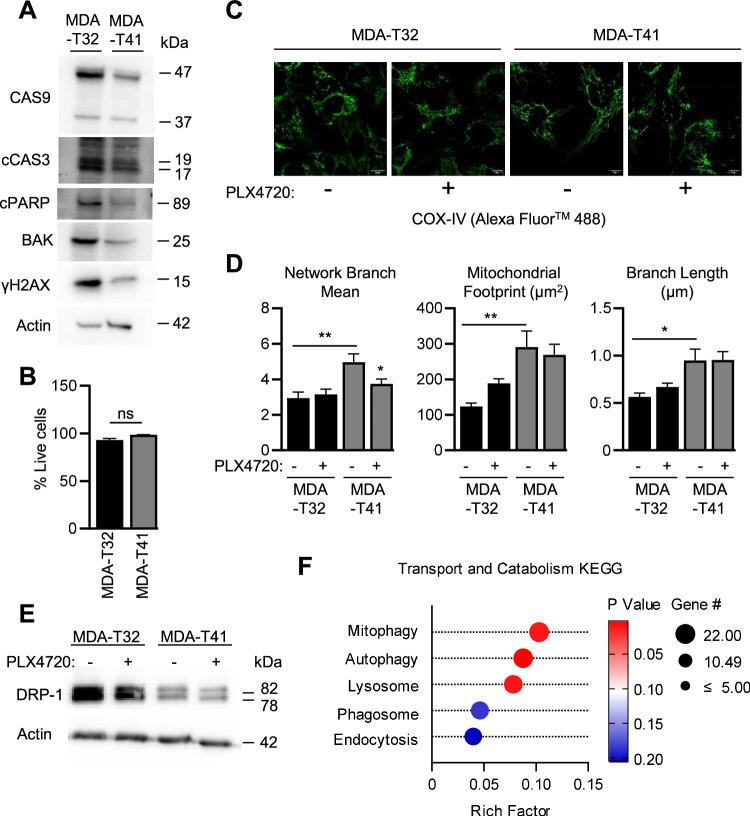
MDA-T32 exhibits failed apoptosis hallmarks. **A** Western blot analysis of apoptosis-associated proteins in PTC cell lines at basal conditions. **B** Quantified flow cytometry analysis of 7-aad staining in PTC cells. Data points are representative of the percentages of healthy cells (7-aad^-^) from three independent experiments with error bars signifying ±SEM, *n* = 3. **C** Representative confocal images of PTC cell lines stained with anti-COX-IV under 60× objective after overnight incubation in DMSO or PLX4720 (1000 nM) treatment. **D** Using Mitochondrial Network Analysis (MiNA) toolset to highlight differences in mitochondrial morphology between resistant and susceptible PTC cell lines. Data points are representative of MiNA analysis of at least 12 different cells from three different staining events. Error bars are ±SEM, the * is indicative of *p* < 0.05 as determined by one-way ANOVA analysis with multiple comparisons. **E** Western blot analysis of DRP-1 in PTC cell lines after an overnight incubation with DMSO or PLX4720 (1000 nM). **F** Bubble plot of KEGG transport and catabolism pathway comparing MDA-T32 to MDA-T41 displaying rich factor, *p*-value, and gene #.

Given that qPCR analysis highlights several inflammatory genes (Fig. 2D, E), and mitochondria play a prominent role in inflammatory regulation [44] and apoptotic resistance to BRAFi treatment in mutant BRAF^V600E^ thyroid cancer cell lines [45, 46], we questioned if “failed apoptosis” is contributing to the intrinsic BRAFi resistance of MDA-T32. Failed apoptosis is a cellular state associated with sublethal caspase activation and fragmented mitochondria known to play a role in cancer progression and drug resistance [25, 47]. To test this, we first analyzed differences in apoptotic protein expression and found the resistant MDA-T32 cells have increased basal levels of cleaved PARP and caspases, pro-apoptotic BAK, and DNA damage as indicated by elevated levels of γH2AX (Fig. 3A). Importantly, these elevated apoptotic/cell stress signals are not sufficient to cause cell death (Fig. 3B) We next analyzed mitochondrial morphology and found MDA-T32 presented with small, circularized mitochondria, while MDA-T41 had longer, fused networks (Fig. 3C, D). These differences persisted in the context of BRAFi. Additionally, western blot analysis indicates DRP-1, a GTPase involved in mitochondrial fission and mitophagy [48], was elevated in MDA-T32 cells (Fig. 3E), an observation that may be linked to the smaller mitochondrial size (Fig. 3C, D) and contributes to failed apoptosis [27]. Further analysis of transcriptomic data [40] highlights enriched KEGG pathways relating to transport and catabolism. Notably, mitophagy and autophagy – cellular processes known to be associated with fragmented mitochondria and failed apoptosis [49] are upregulated in MDA-T32 (Fig. 3F).

### MDA-T32 are reliant on Mcl-1 and exhibit a minority MOMP-like phenotype

**Fig. 4:**
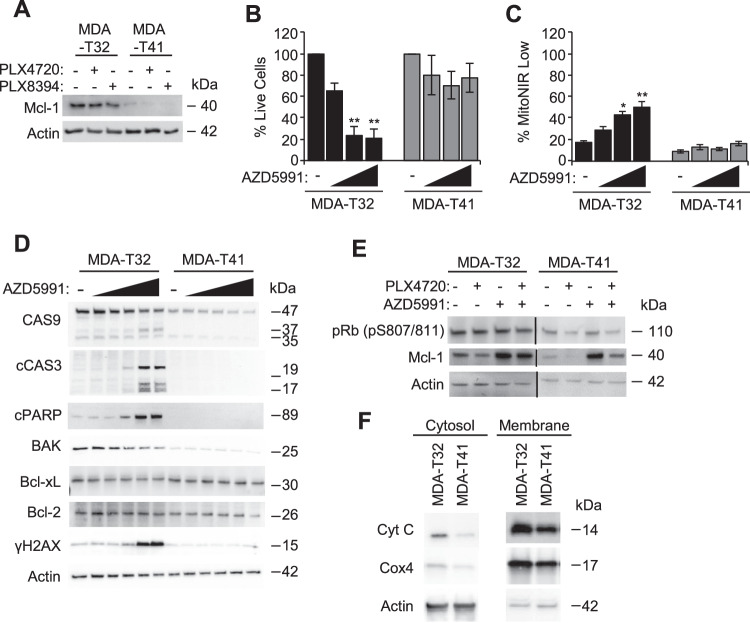
MDA-T32 is reliant on Mcl-1. **A** Western blot analysis of Mcl-1 in PTC cell lines after 24-h treatment with DMSO, PLX4720 (1000 nM), or PLX8394 (1000 nM). **B**, **C** Quantified Flow cytometry analysis of AnnexinV/7-aad (**B**) or MitoNIR (**C**) staining in PTC cells after an overnight incubation with DMSO or AZD5991 (1000 nM, 2000 nM, or 5000 nM). Data points are representative percentages of healthy cells (**B**) or unhealthy mitochondria (**C**) from three independent experiments with error bars signifying ±SEM, *n* = 3. The * is indicative of *p* < 0.05, ** of *p* < 0.01, *** of *p* < 0.001, and # *p* < 0.0001 as determined by one-way ANOVA analysis with multiple comparisons. **D** Western blot analysis of select failed apoptosis-associated proteins in PTC cell lines after a 4-h incubation with DMSO or AZD5991 (1 nM, 10 nM, 100 nM, 1000 nM, or 2000 nM). **E** Western blot analysis of Rb phosphorylation and Mcl-1 stabilization in PTC cell lines after a 24-h incubation with DMSO, PLX4720 (1000 nM), or AZD5991 (2000 nM or 5000 nM). **F** Western blot analysis of cell fractionation comparing cytosolic cytochrome c in MDA-T32 and MDA-T41.

Expression of pro-survival Bcl-2 family members in MDA-T32 could be responsible for maintaining viability in the presence of inflammatory signaling (Fig. 2C–E), sublethal caspase activity (Fig. 3A), and mitochondrial dysfunction (Fig. 3C, D). Mcl-1 is a Bcl-2 family member known to correlate with PTC aggressiveness and resistance to BRAFi [50], and can regulate the clearance of dysfunctional mitochondria [51]. Western blot analysis revealed higher Mcl-1 expression in MDA-T32 compared to the susceptible MDA-T41 cell line and was maintained when challenged with BRAFi (Fig. 4A). We found this increased expression of Mcl-1 to be critically important to MDA-T32 survival as pharmacological inhibition with the BH3 mimetic, AZD5991 [52], strongly elicited cell death in MDA-T32 and not MDA-T41 (Fig. 4B), suggesting Mcl-1 dependency. Cell death in MDA-T32 is likely an on-target effect as AZD5991 induces a concentration-dependent loss of mitochondrial membrane potential (Fig. 4C). Furthermore, we found that only MDA-T32 cells treated with increasing concentrations of AZD5991 associates with elevated caspase and PARP cleavage, and higher levels of DNA damage (Fig. 4D). These results were consistent across acute and later time points (Supplemental Fig. 4A, B). It is noteworthy that the combination treatment of BRAFi (PLX4720) and AZD5991 reveals that Mcl-1 inhibition is likely cytotoxic to MDA-T32 cells as it did not reduce Rb phosphorylation (Fig. 4E).

Minority mitochondrial outer membrane permeabilization (minority MOMP) is a component of sublethal apoptotic signaling oftentimes presenting in failed apoptosis that can be reliant on Mcl-1 activity [53]. While mitochondrial membrane permeabilization and the release of cytochrome c into the cytosol is a hallmark of canonical apoptotic cell death, in minority MOMP, cytochrome c can be released at sublethal levels and stimulate stress responses known to contribute to therapy resistance [54]. Therefore, we queried the levels of cytosolic cytochrome c in MDA-T32 and MDA-T41. Cellular fractionation demonstrated increased basal cytosolic cytochrome c in drug-resistant MDA-T32 compared to susceptible MDA-T41 (Fig. 4F), further highlighting a minority MOMP-like phenotype [24, 53].

Additionally, since autophagic and lysosomal signatures are upregulated in MDA-T32 (Fig. 3F), and autophagy is associated with minority MOMP [49] we investigated autophagic properties of MDA-T32. We found increased molecular weight of LAMP-1 in MDA-T32, likely indicating enhanced glycosylation used to stabilize lysosomes in stressed conditions [55] (Supplemental Fig. 5A). Furthermore, treatment with AZD5991 reduced lysosomal acidification, suggesting lysosomal membrane permeabilization (LMP) (Supplemental Fig. 5B, C). LMP converges with MOMP in the cell death cascade [56, 57]. Together, these observations support the notion that MDA-T32 exhibits a Mcl-1 dependent minority MOMP-like phenotype that may provide resistance to BRAFi.

### Failed apoptosis reduces sensitivity to BRAFi

**Fig. 5:**
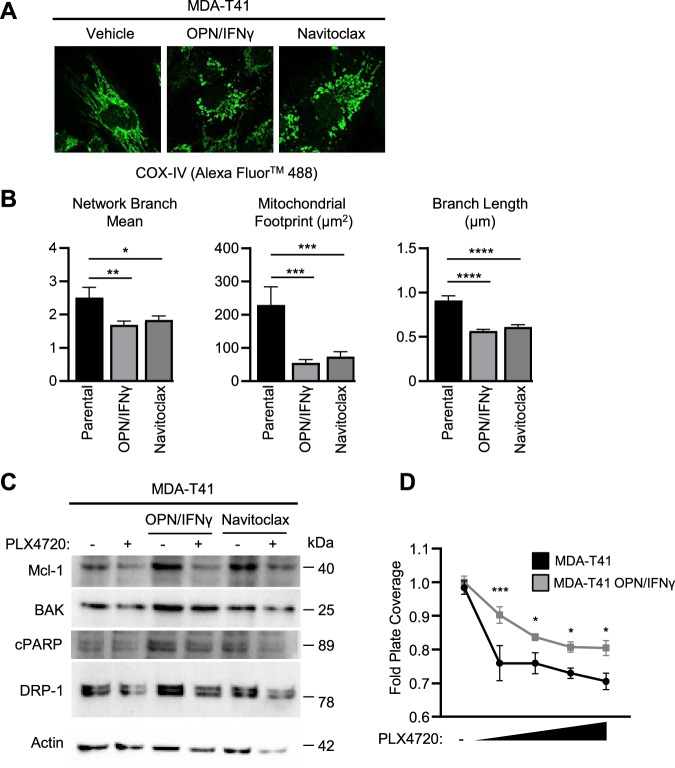
OPN/IFNγ and Navitoclax elicit features of a minority MOMP-like phenotype in MDA-T41. **A** Representative confocal images of parental MDA-T41 cells, MDA-T41 cells habitually treated with OPN (100 ng/mL) and IFNγ (20 ng/mL) for 7 days, or MDA-T41 cells habitually treated with Navitoclax (10 nM) for 7 days prior to being stained with anti-COX-IV, imaged under 60× objective. **B** Quantification of mitochondrial morphology using Mitochondrial Network Analysis (MiNA) toolset to highlight differences between habitually treated MDA-T41 cells. Data points are representative of MiNA analysis of at least 9 different cells from three different staining events. **C** Western blot analysis of failed apoptosis-associated proteins in MDA-T41 cells with or without habitual treatment with OPN (100 ng/mL) and IFNγ (20 ng/mL) or Navitoclax (10 nM). Cells were treated with DMSO or PLX4720 (1000 nM) for 24 h. **D** Quantification of crystal violet staining of parental MDA-T41 cells versus MDA-T41 cells chronically exposed to OPN and IFNγ after 6 days treated with increasing doses of PLX4720 (100 nM, 250 nM, 500 nM, and 1000 nM), represented by fold plate coverage compared to DMSO treatment. Error bars are ±SEM, the * is indicative of *p* < 0.05, ** of *p* < 0.01, *** of *p* < 0.001, and **** *p* < 0.0001 as determined by one-way ANOVA (**B**) and two-way ANOVA analysis (**D**) with multiple comparisons (**B**, **D**).

Failed apoptotic cell states including sublethal caspase activation and impaired mitochondrial membrane integrity can promote pro-inflammatory stress signaling loops [53, 58]. As a proof-of-concept, we questioned if the failed apoptosis phenotype found in the MDA-T32 cells can be replicated in the susceptible MDA-T41, thereby affording resistance to BRAFi. To explore this, we habitually treated MDA-T41 cells with OPN and IFNγ (inflammatory mediators found to be upregulated in MDA-T32 (Fig. 2D, E)). In parallel, we treated MDA-T41 with low doses of the BH3 mimetic, Navitoclax, to directly induce mitochondrial permeabilization. While these treatments were sublethal (Supplemental Fig. 6A), prolonged exposure to OPN/IFNγ or Navitoclax in MDA-T41 cells resulted in altered mitochondrial morphology (Fig. 5A, B) and protein expression signatures consistent with failed apoptosis (Fig. 5C). Importantly, we found that habitual treatment of MDA-T41 cells with OPN and IFNγ resulted in elevated levels of Mcl-1 and correlated with reduced PLX4720 sensitivity (Fig. 5D). Interestingly, while low dose navitoclax treated cells presented with fragmented mitochondria and elevated Mcl-1, it did not provide resistance to BRAFi (Supplemental Fig. 6B) suggesting that directly targeting mitochondria alone is not capable of providing failed apoptosis-associated therapy resistance. These data further support that failed apoptosis and mitochondrial dysfunction can contribute to BRAFi resistance and are at least in part associated with inflammatory mediators.

### PI3K/AKT inhibition sensitizes resistant MDA-T32 cells to BRAFi

**Fig. 6:**
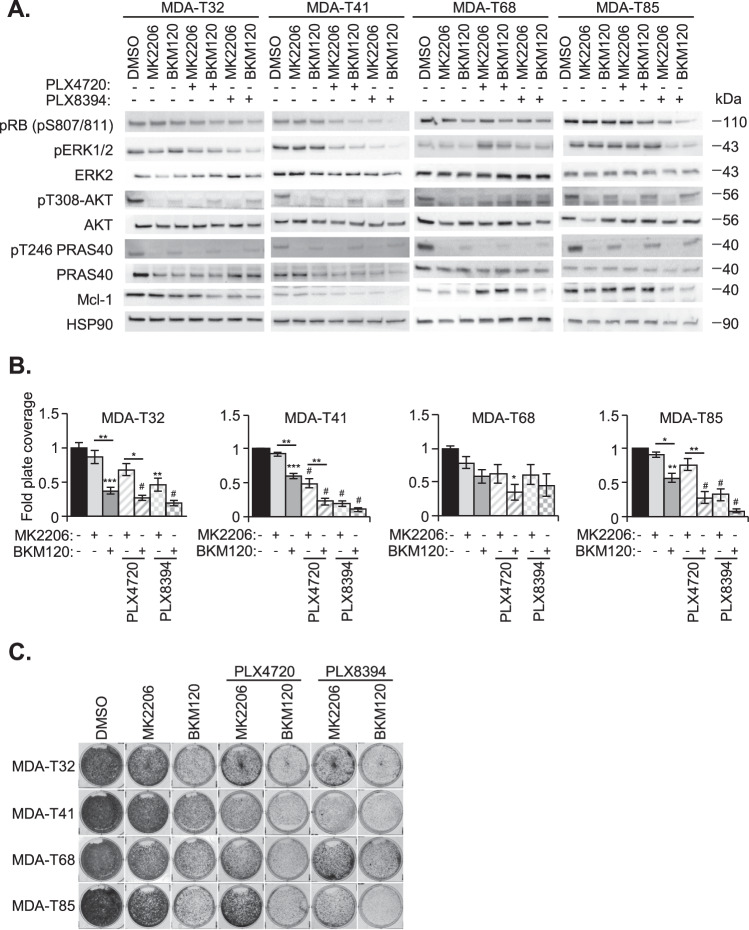
PI3K/AKT inhibition targets Mcl-1 dependency. **A** Western blot analysis of PTC cell lines treated with monomer selective BRAFi and/or PI3K/AKT inhibitors. Cells were treated for 24 h with DMSO, AKT inhibitor MK2206, PI3K inhibitor BKM120, or either therapies in combination with PLX4720 or PLX8394. Lysates were probed with the indicated primary antibodies. **B** Fold plate coverage of crystal violet staining fixed proliferating PTC cells after a 6-day incubation in DMSO, MK2206, BKM120, or either therapy in combination with PLX4720 or PLX8394. The error bars are ±SEM (**B**). The * is indicative of *p* < 0.05, ** of *p* < 0.01, *** of *p* < 0.001, and # *p* < 0.0001 as determined by one-way ANOVA analysis with multiple comparisons (**B**). **C** Representative crystal violet staining of PTC cells after a 6-day incubation in DMSO MK2206, BMK120, or combination treatments with PLX4720 or PLX8394.

Since Mcl-1 inhibitors have struggled in clinical trials [59], and expression of Mcl-1 is regulated by ERK1/2 [60] and AKT signaling [61] we questioned if combined treatment of BRAFi with AKT or PI3K inhibitors would be efficacious against MDA-T32. We found that the AKT inhibitor, MK2206, and PI3K inhibitor, BKM120, effectively blocked AKT activation, reduced phosphorylation of downstream targets like PRAS40, and modestly reduced Mcl-1 levels in all cell lines (Fig. 6A). Notably, while BRAFi combinations with MK2206 or BKM120 reduced growth of all mutant BRAF PTC cell lines, BKM120 monotherapy did not reduce ERK1/2 activity or Rb phosphorylation, yet suppressed growth as indicated by crystal violet staining (Fig. 6B, C). Overall, combined inhibition of the AKT pathway with BRAFi treatment may overcome Mcl-1-mediated innate resistance in mutant BRAF PTC.

### Mutant BRAF PTC patient samples demonstrate minority MOMP-associated resistance

**Fig. 7:**
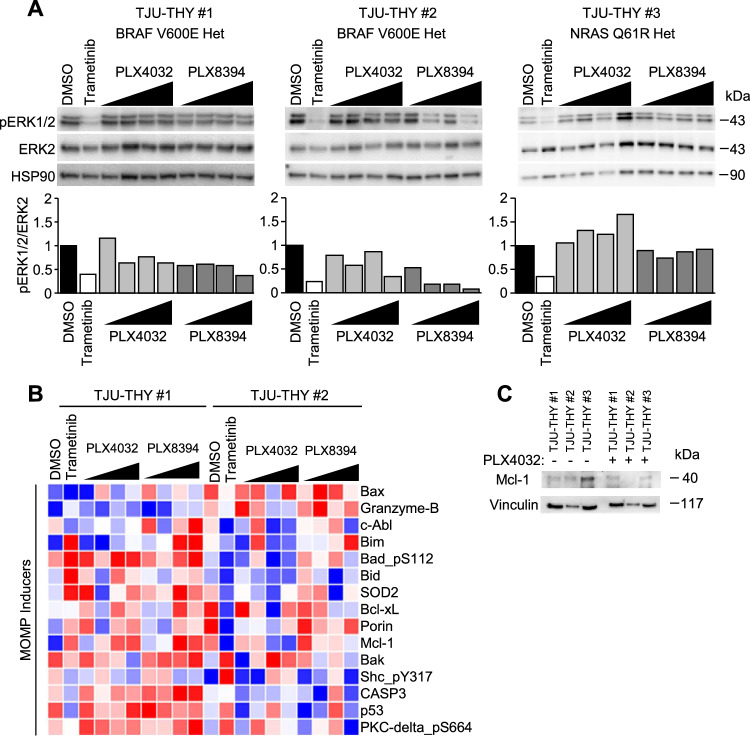
Ex vivo analysis of PTC patient samples reveals minority MOMP-like patterns. **A** ~1–2 mm pieces of freshly resected PTC tissue samples were incubated with DMSO, trametinib (50 nM), or increasing concentrations (100 nM, 500 nM, 1000 nM, 5000 nM) of PLX4032 or PLX8394 for 48 h. Lysates were harvested, and samples were probed via western blot analysis. Quantification of phosphorylated ERK1/2 (pERK1/2) to ERK2 as determined by densitometry is graphed below. **B** RPPA protein targets represented in the mitochondrial permeabilization gene set [63] are shown. **C** Western blot analysis of Mcl-1 in PTC patient samples incubated with and without PLX4032 (1000 nm) for 24 h.

To assess the clinical relevance of the MDA-T32 resistant phenotype, we utilized our previously established patient-derived explant system (PDeX) [30]. In PDeXs, freshly isolated surgical tissue is directly assayed in an ex vivo culture system (Supplemental Fig. 7A). We obtained three PTC samples and performed Sanger sequencing of BRAF and NRAS – common oncogenic mutations in PTC [62]. Two samples – TJU-THY #1 and #2 were wildtype for NRAS and have heterozygous mutation for BRAF^V600E^ (Supplemental Fig. 7B). In contrast, TJU-THY #3 has a heterozygous NRAS^Q61R^ mutation and is wildtype for BRAF (Supplemental Fig. 7B). In parallel to sequencing analysis, fresh samples were divided into small (~1–2 mm) pieces and placed on a gelatin based hemostatic sponge soaked in culture media with DMSO, trametinib, the clinical grade BRAFi vemurafenib (PLX4032 – analogous to PLX4720), and PLX8394. Samples were incubated ex vivo for 48 h and processed for western blotting. Results indicate that while trametinib inhibited ERK1/2 phosphorylation across all three PTC samples, BRAFi induced heterogeneous responses (Fig. 7A). PLX4032 had modest effects on the mutant BRAF TJU-THY #1 and #2 and elicited dose-dependent paradoxical ERK1/2 activation in the wildtype BRAF sample TJU-THY #3; an effect similar to the response of BRAF wildtype MDA-T68 (Fig. 1). PLX8394 inhibited ERK1/2 signaling in TJU-THY #2 but had modest effects in TJU-THY #1. Furthermore, as expected, PLX8394 had little effect on the wildtype BRAF TJU-THY #3.

We employed reverse phase protein array (RPPA) analysis to better understand how the patient samples respond to drug treatment. Hierarchical clustering highlights the differences between the mutant BRAF samples and mutant NRAS (Supplemental Fig. 7C). While there was large overlap between TJU-THY #1 and #2, sections of divergent expression also exist (Supplemental Fig. 7C). Given the more refractory nature of TJU-THY #1 (Fig. 7A), we questioned if this sample possessed intrinsic resistance paralleling MDA-T32. To this end, protein targets in the RPPA dataset present in the curated Mitochondrial Permeability [63] pathway are highlighted (Fig. 7B). In addition, western blot analysis indicates TJU-THY #1 maintains Mcl-1 expression when challenged with the BRAFi PLX4032 (Fig. 7C), paralleling MDA-T32 (Fig. 4A). It is noteworthy that while increased levels of MOMP inducers potentially affords minority MOMP-like mediated drug resistance, this was not directly assayed in the patient samples.

## Discussion

The current standard of care for advanced papillary thyroid cancer is surgical resection followed by RAI and HRT [3]. However, since 80% of thyroid cancer patients are diagnosed at a low median age, patients are subjected to prolonged exposure to HRT [3]. Extended periods of treatment increase the risk of adverse effects such as secondary cancers [5], cardiovascular issues, and endocrine dysfunction [64]. Highlighting a need for improved treatments to selectively eliminate malignant tissue while salvaging healthy tissue for improved quality of life for PTC patients.

Originally thought to be an inflection point in the apoptotic process, sublethal mitochondrial outer membrane permeability (MOMP) has recently been observed as a chronic phenomenon associated with apoptotic resistance dependent on expression of pro-survival Bcl-2 members [23, 24, 26, 27, 53]. While the direct role of anti-apoptotic Bcl-2 proteins in facilitating minority MOMP is not entirely understood, here we demonstrate the importance of Mcl-1 in a minority MOMP-like phenotype that may support innate BRAFi resistance in mutant BRAF PTC cells.

Initial observations indicate our PTC cell line panel (MDA-T32, -T41, -T68, and -T85) [28] have heterogeneous responses to BRAFi treatment (Fig. 1). Even though they share similar mutational profiles, MDA-T32 exhibits innate resistance to BRAFi while MDA-T41 is sensitive. Transcriptional profiling highlights increased expression of gene targets associated with inflammatory signaling and stress response in MDA-T32. It is noteworthy that while these pathways are associated with MOMP [24, 26, 47], they were also recently highlighted in a scRNA-seq-based study in melanoma that identified many of the MDA-T32 associated pathways in a subpopulation of BRAFi “escapees” – likely representing intrinsically resistant melanoma [65]. Transcriptomic analysis also revealed MDA-T32 had high levels of VCAM1 (Supplemental Fig. 3), a protein known to contribute to PTC aggressiveness and therapy resistance [42, 43]. While our experiments did not indicate a specific role for VCAM1 in BRAFi resistance, its expression contributed to the enrichment score of Epithelial to Mesenchymal Transition and Apical Junction pathways (Fig. 2C) – both known to dictate prognosis and drug sensitivity [66, 67].

We identified signatures of failed apoptosis and smaller, more circularized mitochondria in the resistant MDA-T32 cells – correlating with increased expression of the mitochondrial fission regulator, DRP-1 (Fig. 3). Sublethal stressors can induce mitochondrial dysfunction and modulate fission and fusion patterns across networks [24]. Increased mitochondrial fission through DRP-1 can directly influence DNA damage and apoptotic threshold to facilitate minority MOMP [27, 68]. Furthermore, mitochondrial dysfunction can induce low-level release of cytochrome c and other stress signals prolonging/stabilizing the minority MOMP phenotype [24, 69]. Notably, we found MDA-T32 exhibits higher levels of cytosolic cytochrome c compared to that of susceptible MDA-T41 (Fig. 4F); an effect which may be linked to the elevated levels of DNA damage (Fig. 4A) and together provide evidence of a minority MOMP-like phenotype [24, 26].

The ability of cancer cells to evade the apoptotic effects of BH3 mimetics has previously been attributed to stress-induced minority MOMP [54]. Here, we use low doses of the pan-BH3 mimetic, Navitoclax, to promote mitochondrial dysfunction in MDA-T41. Navitoclax treatment altered mitochondrial morphology and elicited protein signatures similar to failed apoptosis patterns observed in MDA-T32 (Fig. 5A–C). Furthermore, we cultured MDA-T41 cells with inflammatory mediators upregulated in MDA-T32 (OPN and IFNγ) and found increased expression of failed apoptosis hallmarks and reduced sensitivity to BRAFi (Fig. 5). This suggests that inflammatory mediators can drive failed apoptosis and can at least in part contribute to therapeutic resistance in PTC. In contrast, while directly inducing mitochondrial dysfunction via navitoclax treatment conferred some aspects of failed apoptosis (Fig. 5A–C), it did not decrease sensitivity to BRAFi (Supplemental Fig. 6B) – potentially indicating failed apoptosis-associated BRAFi resistance requires broader inflammatory signaling.

While some aspects of minority MOMP have been identified in mutant BRAF thyroid carcinoma [46], the role of Mcl-1 in this process is not well understood. Mcl-1 expression is critical to maintaining minority MOMP in esophageal cancer models [53], but this function has yet to be highlighted in other cancers. In our present study, we illustrate how MDA-T32 cells are reliant on Mcl-1 function as Mcl-1 inhibitor treatment induces hallmarks of complete MOMP, associating with cell death (Fig. 4). In addition, our data suggest Mcl-1 may also maintain autophagy and lysosomal homeostasis in MDA-T32 cells (Supplemental Fig. 5). These observations parallel studies demonstrating a role for Mcl-1 in regulating autophagy upon mitochondrial stress [51], and others linking lysosomal membrane permeabilization with MOMP [56, 57].

Considering that Mcl-1 inhibitors have struggled to induce tumor regression in the clinic [59], and since the MAPK and PI3K/AKT pathways are active in aggressive thyroid cancer [70, 71] and can regulate Mcl-1 expression [60, 61], we questioned if targeting the PI3K/AKT axis is efficacious against Mcl-1 dependent mutant BRAF PTC. When treated with PI3K/AKT inhibitors in combination with BRAFi, MDA-T32 had reduced AKT signaling and Mcl-1 levels, correlating with slowed cell growth (Fig. 6). These data may support clinical findings highlighting the benefits of drugging multiple targets [72, 73] over individual signaling pathways [22, 74].

**Fig. 8:**
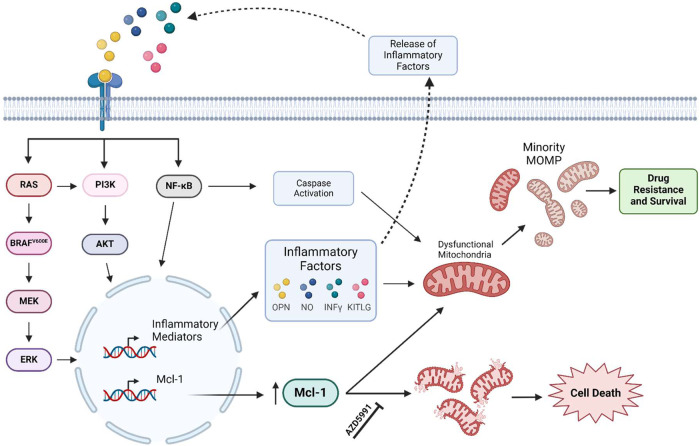
Inflammation-mediated minority MOMP-like drug resistance is dependent on Mcl-1. Schematic overview of the proposed innate resistance mechanism of MDA-T32 mutant BRAF^V600E^ PTC cell line. Mitogenic signaling stimulates the expression of inflammatory mediators and Mcl-1. Inflammatory signaling likely drives feed-forward activity and in parallel triggers minority MOMP-like mitochondrial stress. Elevated Mcl-1 levels are required to maintain sufficient mitochondrial viability in the inflamed, drug-resistant state.

Taken together, we demonstrate that mutant BRAF PTC has heterogeneous responses to BRAFi. We found MDA-T32 to be intrinsically resistant to BRAFi, an effect likely associated with increased inflammatory signaling. In line with this, we show MDA-T32 has hallmarks of failed apoptosis/minority MOMP and are acutely dependent on Mcl-1 expression to counterbalance aberrant MOMP and cell death (Fig. 8). Additionally, we found combined treatment with PI3K/AKT inhibitors sensitizes MDA-T32 to BRAFi – an effect potentially associated with reduced Mcl-1 expression. Lastly through ex vivo analysis, we identified a freshly biopsied patient sample that exhibits innate resistance to BRAFi and molecular signatures similar to MDA-T32 (Fig. 7). While we acknowledge proteomic analysis of these patient samples could be skewed by components of the tumor immune microenvironment, BRAFi resistance may at least in part be attributed to a Mcl-1 regulated minority MOMP-like phenotype (Fig. 7), paralleling our observations of MDA-T32. Taken together, our study may point to failed apoptosis and minority MOMP as a mechanism underpinning poor efficacy and response rates of BRAFi-treated mutant BRAF PTC patients.

## METHODS

### Cell culture

MDA-T32, -T41, -T68, and -T85 were all purchased from American Type Culture Collection (ATCC) in December 2018. Cells were grown in RPMI-1640 (ATCC 30-2001™) supplemented with 10% FBS, 1% NEAA, and 1% L Glutamine (except MDA-T85 – as directed by ATCC). Cell lines were utilized at low passage and were routinely assayed for mycoplasma contamination with MycoScope Kit (Genlantis, San Diego, CA).

### Inhibitors and antibodies

Trametinib (GSK1120212) (#S2673), PLX4720 (#S1152), PLX4032 (#S1267), PLX8394 (#S7965), AZD5991 (#S8643), MK2206 (#S1078), buparlisib (BKM120) (#S2247), and Navitoclax (#S1001) were purchased from Selleck Chemicals, LLC. DRP-1 (#8570S), BAK (#12105S), Bcl-XL (2762S), Caspase 9 (#9502S), Cleaved caspase 3 (#9661S), HSP90 (#4877S), pERK1/2 (#9101S), pRb S807/811 (#9308S), pT308 AKT (#2965S), pPRAS40 T246 (#2640S), VCAM1 (#13662S), Mcl-1 (#4572S), LAMP-1 (#9091T), Vinculin (#4650S), Tubulin (#2148S), COX-IV (#11967S), cytochrome c (#11940S) cleaved PARP (#9541) and GAPDH (#2118S) antibodies were purchased from Cell Signaling Technology. ERK2 (sc-1647) and AIF (#sc-13116) were purchased from Santa Cruz Biotechnology Inc., γH2AX (ab11174) was purchased from Abcam, Actin (A2066) was purchased from Sigma-Aldrich Co., and Bcl-2 (#51-6511GR) was purchased from BD Biosciences. Goat anti-rabbit IgG Alexa Fluor^TM^ 488 secondary antibody was purchased from Invitrogen.

### Lentiviral cloning and stable cell lines

Human VCAM1 was amplified from cDNA (Dharmacon Clone Id: 30339170), cloned into pENTR/D-TOPO entry vectors (Invitrogen), and recombined into pLenti4.3/TO/DEST using LR Clonase II (Invitrogen). 3 μg of pLenti4.3/TO/huVCAM-1 and appropriate lentiviral packaging plasmids were transfected into LentiX-293t cells (Takara) for lentiviral production using FuGene6 Transfection reagent (Promega). Viral particles were precipitated after 72 h and transduced into MDA-T41TR cells for inducible expression under doxycycline control. MDA-T41TR were treated with zeocin for antibiotic selection 48 h post-transduction for two weeks. Drug-resistant clones were isolated and used for analysis.

### Ex vivo explant system

Tumors were collected following informed patient consent at Thomas Jefferson University Hospital under an IRB-approved protocol (#07D.483). The following PTC samples were isolated: TJU-THY #1 is from a 53 y/o, TJU-THY#2 is from a 32 y/o female, and TJU-THY#3 is from a 38 y/o. Less than 16 h post-surgery, excess adipose and stromal tissue was removed and tumors were cut into 1 mm^3^ pieces. Vetspon absorbable hemostatic gelatin 1 cm^3^ sponges (Novartis; Basel, Switzerland) were pre-soaked in 12-welled plates for 15 min at 37 °C in 500 μL of DMEM/10% FBS containing drugs or DMSO as a vehicle control. To avoid concerns of intratumoral heterogeneity, up to three 1 mm^3^ pieces from different locations of the original tumor were placed per sponge per treatment condition. Medium was replaced every 24 h. Tumor pieces for western blotting were homogenized in modified RPPA lysis buffer [75] with phosphatase and inhibitors (PhosSTOP and cOmplete tablets Roche, Basel, Switzerland). Laemmli sample buffer was added, and samples were heated at ≥95 °C for 5 min. In parallel, the lysates were sent to MD Anderson and used for reverse phase protein array (RPPA) analysis as previously described [46]. Data was visualized using Morpheus (Broad Institute).

### Antibody array

RayBioTech C-series RTK Phosphorylation array C1 (AAH-PRTK-1-8) was used to assess the active phosphorylation of 71 targets across different thyroid cancer cell lines. MDA-T32 and MDA-T41 cells were grown to 80–90% confluence in 15 cm plates and incubated overnight in DMSO or PLX4720 (1000 nM) before harvesting and lysing as instructed by the manufacturer. 100 μg of cell lysate was processed as per the manufacturer’s instructions. Results were imaged using BioRad’s Chemidoc XRS imaging system and QuantityOne software. Quantification of targets was performed using densitometry comparing the expression of all targets across the membrane normalized to the average of the positive controls.

### Crystal violet growth assays

Cells (5.0 × 10^4^) were seeded in 6-well plates in culture medium overnight. The next day, the medium was supplemented with drugs of interest. Medium and drugs were renewed every 2 days for 6 days. Subsequently, cells were fixed and stained in buffered formalin with 0.2% crystal violet. Plates were then scanned for quantitation using ImageJ.

### Western blotting

For western blot analysis, cells (2.0 × 10^5^) were seeded in 6-well plates in culture medium overnight. The next day, cells were treated with drug of interest for 24 h. Lysates were harvested in 1× Laemelli buffer and 5% BME. SDS-PAGE gels were loaded with 15 μg of protein per lane and transferred to PVDF membranes via TransBlot® Turbo System (BioRad Laboratories). Membranes were blocked in 1% BSA in PBS containing 0.1% Tween (PBST) for 1 h at room temperature. All primary antibodies were brought up in 1% BSA/PBST and incubated overnight at 4 °C. The following day, membranes were washed three times for 15 min each in PBST before incubating in secondary HRP antibodies brought up in 5% nonfat dry milk/PBST. Western blots were developed in SuperSignal™ West Pico Chemiluminescent Substrate (Thermo Scientific) using the ChemiDoc XRS+ imaging system and Quanitity One imaging software (BioRad Laboratories). Densitometry analysis was conducted in ImageJ. Full uncropped western blots are available in Supplementary data file.

### S-phase entry analysis

Cells (2.0 × 10^5^) were seeded in 6-well plates in culture medium overnight. The next day, cells were treated with the drug of interest for 24 h. The thymidine analog, EdU, was then added at a final concentration of 1.0 × 10^4^ nM for the last 6 h. EdU incorporation was measured using the Click-it EdU Alexa Fluor 647 Cytometry Assay Kit (Invitrogen, Carlsbad, CA) as per the manufacturer’s instructions (Molecular Probes). EdU staining was quantified on CytoFLEX Flow Cytometer (Beckman Coulter, Inc.), and data were analyzed with CytExpert software.

### siRNA transfection

Cells (7.5 × 10^4^) were transiently transfected with Dharmacon siRNAs at a final concentration of 25 nmol/L using Lipofectamine RNAiMAX (Invitrogen). Nontargeting control (5′-UGGUUUACAUGUCGACUAA-3′) or VCAM1 (J-013351-06-0005: 5′-CAAAGUUGGCUCACAAUUA-3′, J-013351-07-0005: 5′-GAAGGAUGCGGGAGUAUAU-3′) siRNAs were incubated with RNAiMAX in OptiMEM (Gibco) at RT for 15 min before administering to cells. Transfection was incubated for 4 h at 37 °C with 5% CO_2_. Serum-containing media was added to the transfection cocktail and incubated for 72 h before experimentation.

### Statistical analysis

Unless noted otherwise, experiments were repeated 3 times and significance was determined by one-way ANOVA analysis with post-hoc Tukey HSD test using GraphPad Prism 10. Data represents mean values and error bars denote the standard error of the mean (SEM). Unless specified, * is indicative of *p* < 0.05, ** of *p* < 0.01, *** of *p* < 0.001 and # *p* < 0.0001.

### Qiagen RT2 qPCR array

A confluent 10 cm plate (2.0 × 10^6^ cells) was treated with the drug of interest overnight before RNA extraction. RNA was extracted using the QIAshredder (Qiagen #79654) and the RNeasy Mini Kit (Qiagen #74104) as per the manufacturer’s instructions. RNA was then converted to cDNA using the iScript cDNA synthesis kit (BioRad #1708891). A human Cancer Inflammation and Immunity Crosstalk (CIIT) (#PAHS-181Z) RT^2^ qPCR array from Qiagen was used. The three replicate arrays were performed utilizing Sybr Green qPCR Mastermix and were analyzed using the analysis spreadsheet provided by Qiagen.

### Mitochondrial viability and lysosomal acidification experiments

Cells (1.5 × 10^5^) seeded in a 6-well plate overnight were treated with 1000, 2000, or 5000 nM AZD5991 for 24 h before being trypsinized and washed with 1% BSA/PBS. 5 μL of 200× MitoNIR reagent from Abcam (ab112149) or 1000 nM of LysoSensor^TM^ green DND-189 (#L7535) from Invitrogen were then added for and incubated in the dark for 30 min. After the incubation, cells were washed and resuspended in 1% BSA/PBS. These samples were then analyzed using a CytoFLEX Flow Cytometer and data were analyzed with CytExpert software and FlowJo.

### Immunofluorescence

Cells were seeded overnight at 2.5 × 10^5^ in glass bottom dishes and treated the following day with DMSO or PLX4720 (1000 nM) for a 24-h incubation. At the endpoint, cells were either fixed and permeabilized immediately for an overnight incubation with COX-IV primary antibody at 4 °C or stained with 500 nM of MitoTracker Green or Red FM (Invitrogen^TM^ M7154, M22425) before fixation with 4% paraformaldehyde. Cells incubated with COX-IV primary were washed and incubated for 1 h at room temperature the following day with Alexa Fluor^TM^ 488 secondary antibody. Images were obtained using a 60× objective on an Olympus Fluoview FV3000 confocal microscope. Mitochondrial morphology was analyzed using the mitochondria network analysis (MiNA) toolset [76].

### Habitual MDA-T41 treatment with OPN/IFNγ or Navitoclax

Cells were seeded (5 × 10^5^) in a 10 cm plate and cultured with 100 ng/mL OPN (#557102) and 20 ng/mL IFNγ (Biolegend #570202) or with 10 nM Navitoclax for 6–21 days prior to use in experiments. Media changes with fresh recombinant protein or navitoclax were performed every other day.

### Cellular fractionation

Cells were seeded (1 × 10^6^) in a 10 cm plate and cultured overnight. The following day, cells were harvested and fractionated with the Cell Fractionation Kit (Cell Signaling #9038) to compare the expression of cytosolic cytochrome c in -T32 and MDA-T41 lines.

### Supplementary information

Supplemental Figure 1

Supplemental Figure 2

Supplemental Figure 3

Supplemental Figure 4

Supplemental Figure 5

Supplemental Figure 6

Supplemental Figure 7

Supplemental Figure Legends

Original Data Files

## Data Availability

The data supporting the findings of this study are available from the corresponding author upon reasonable request.
