# Supplementary figures and images for: Mcl-1 mediates intrinsic resistance to RAF inhibitors in mutant BRAF papillary thyroid carcinoma
Source: Cell Death Discov. 2024 Apr 15;10:175. doi: 10.1038/s41420-024-01945-0 (PMC11018618; doi:10.1038/s41420-024-01945-0)

**A**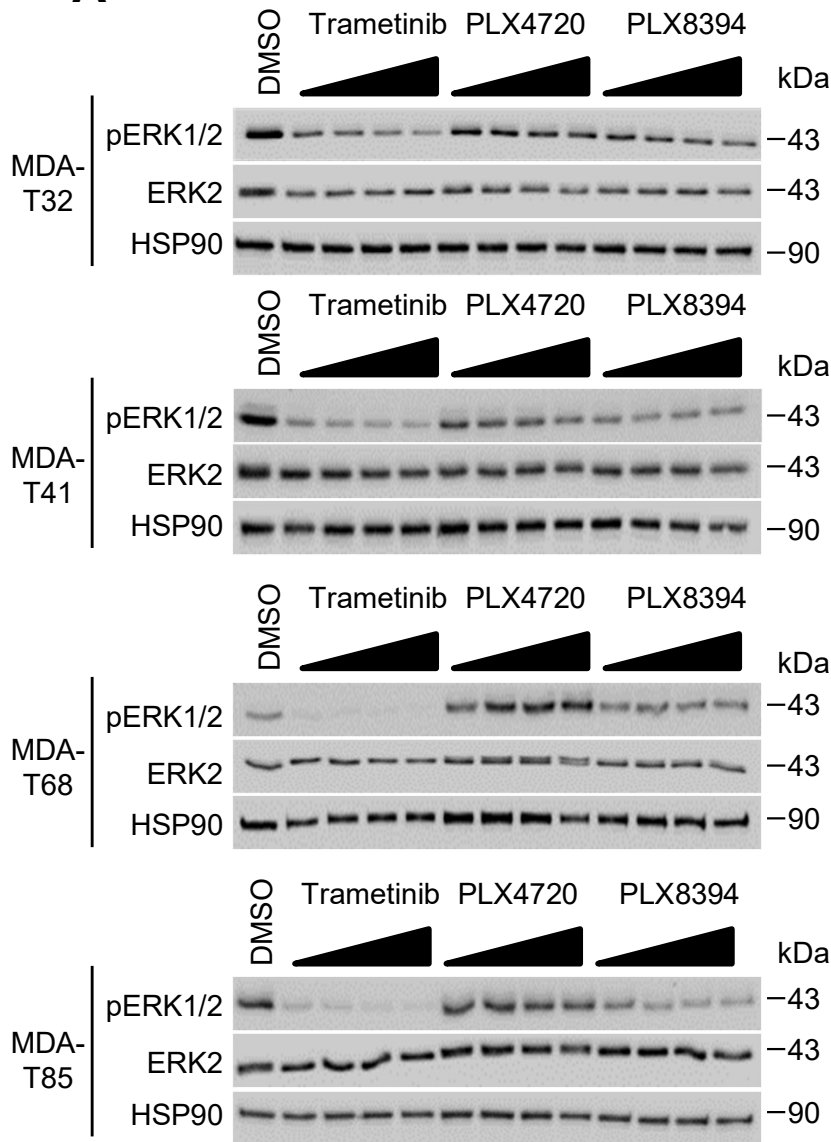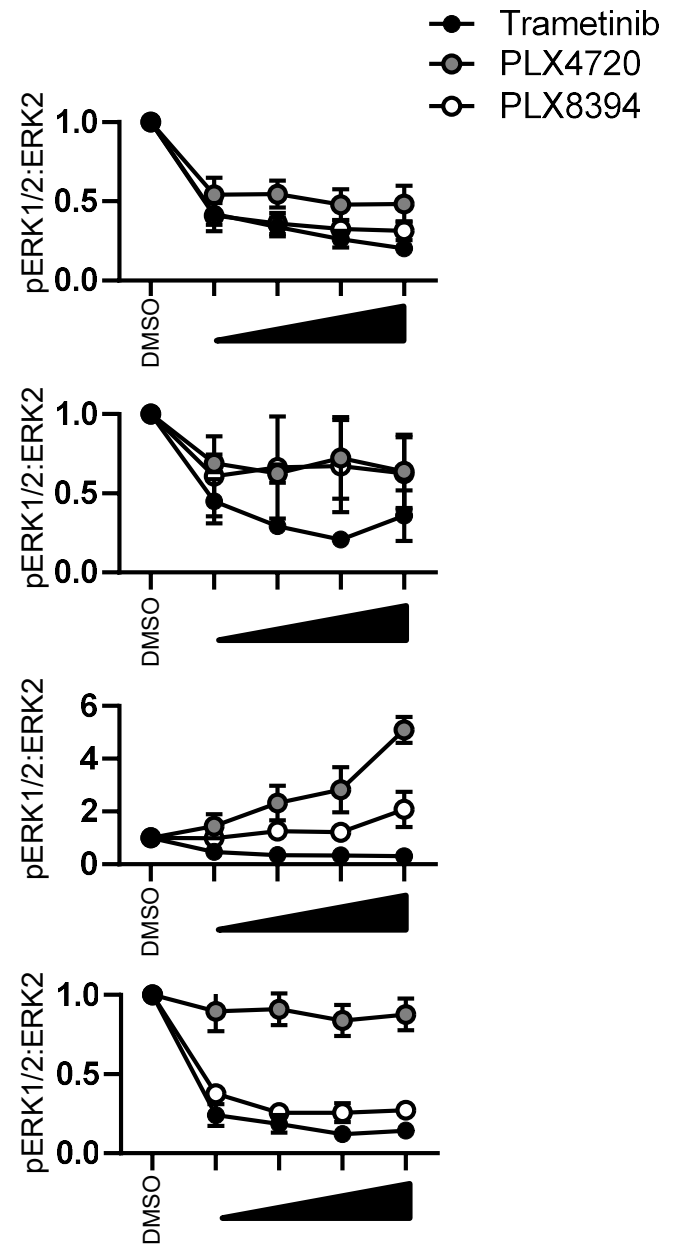

Supplement: Supplementary file 1 — Supplemental Figure 1 [file 41420_2024_1945_MOESM1_ESM.pdf]

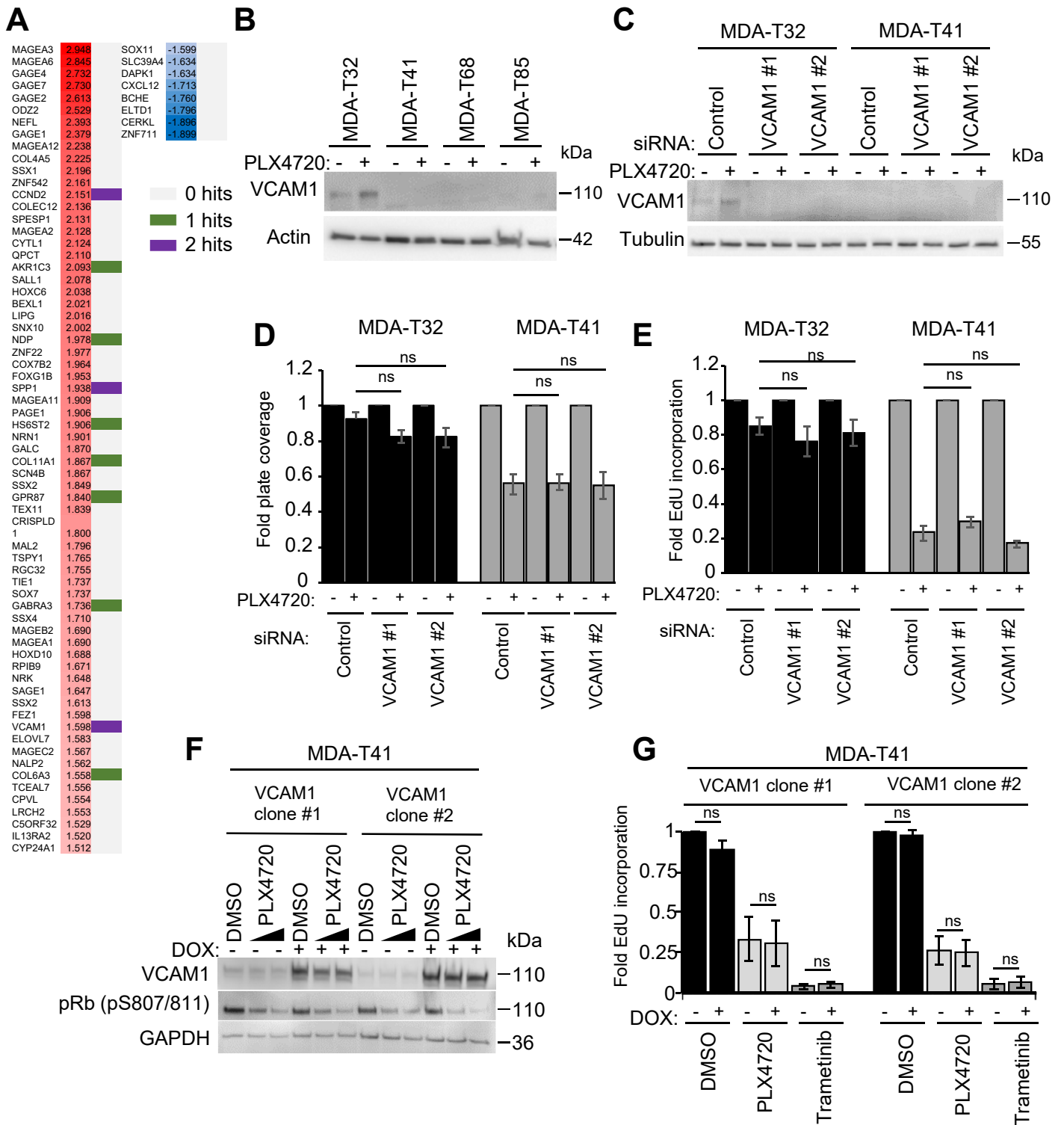

Supplement: Supplementary file 3 — Supplemental Figure 3 [file 41420_2024_1945_MOESM3_ESM.pdf]

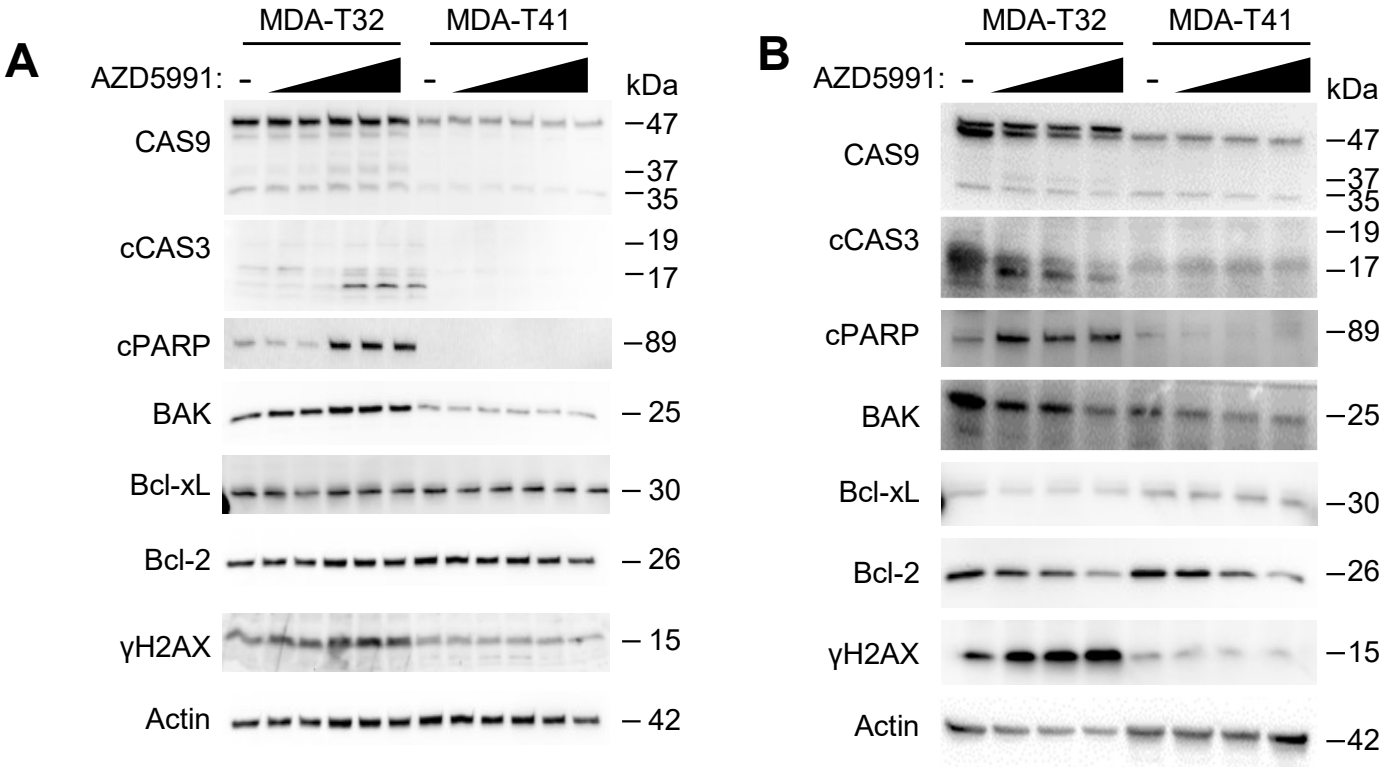

Supplement: Supplementary file 4 — Supplemental Figure 4 [file 41420_2024_1945_MOESM4_ESM.pdf]

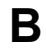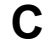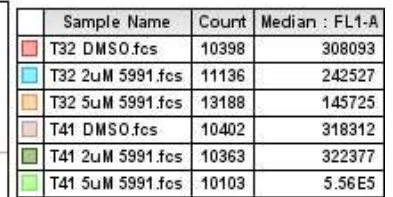

Supplement: Supplementary file 5 — Supplemental Figure 5 [file 41420_2024_1945_MOESM5_ESM.pdf]

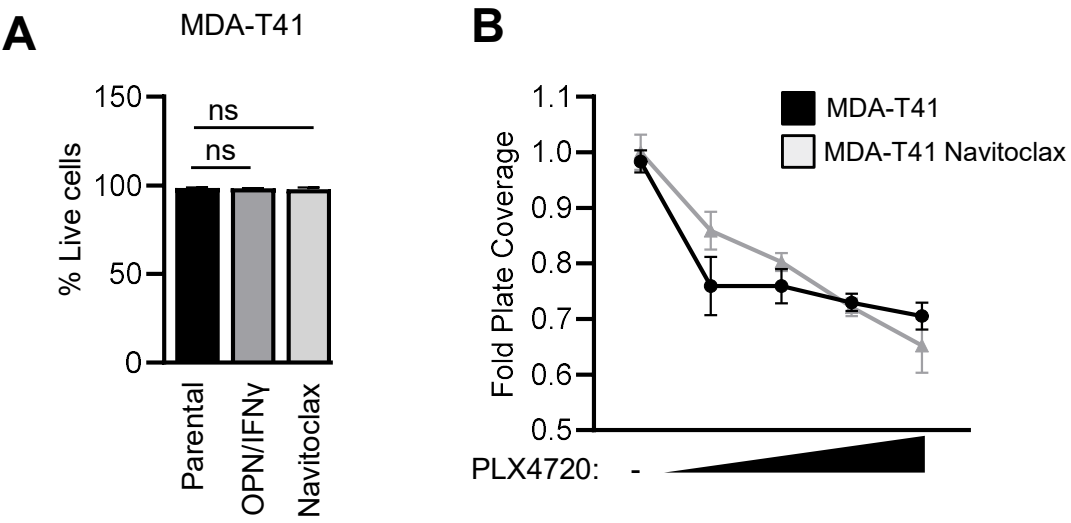

Supplement: Supplementary file 6 — Supplemental Figure 6 [file 41420_2024_1945_MOESM6_ESM.pdf]

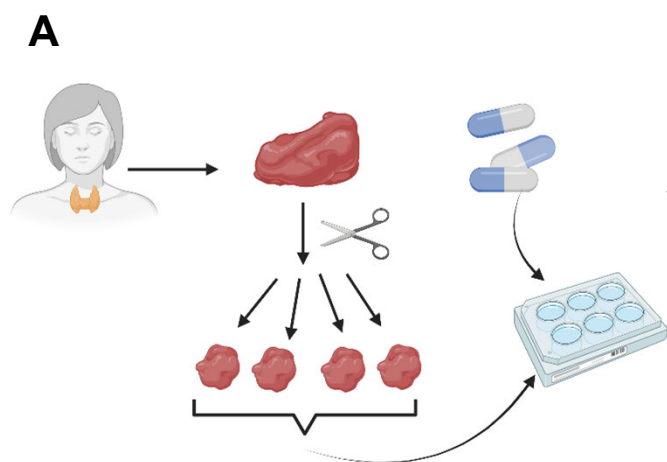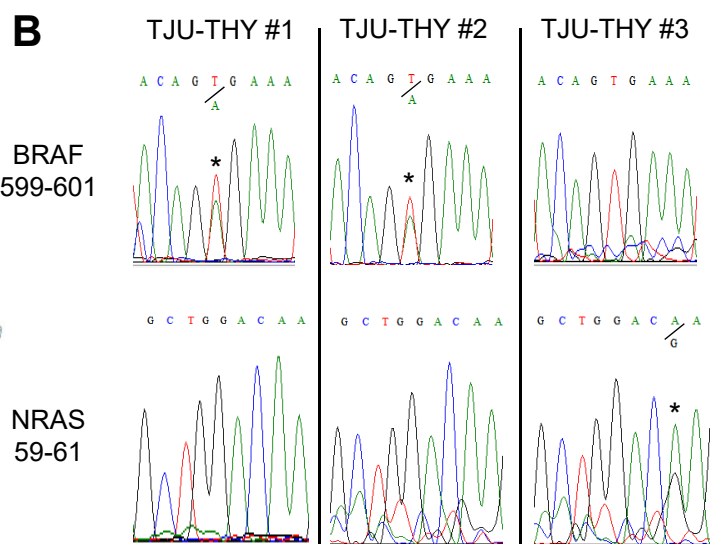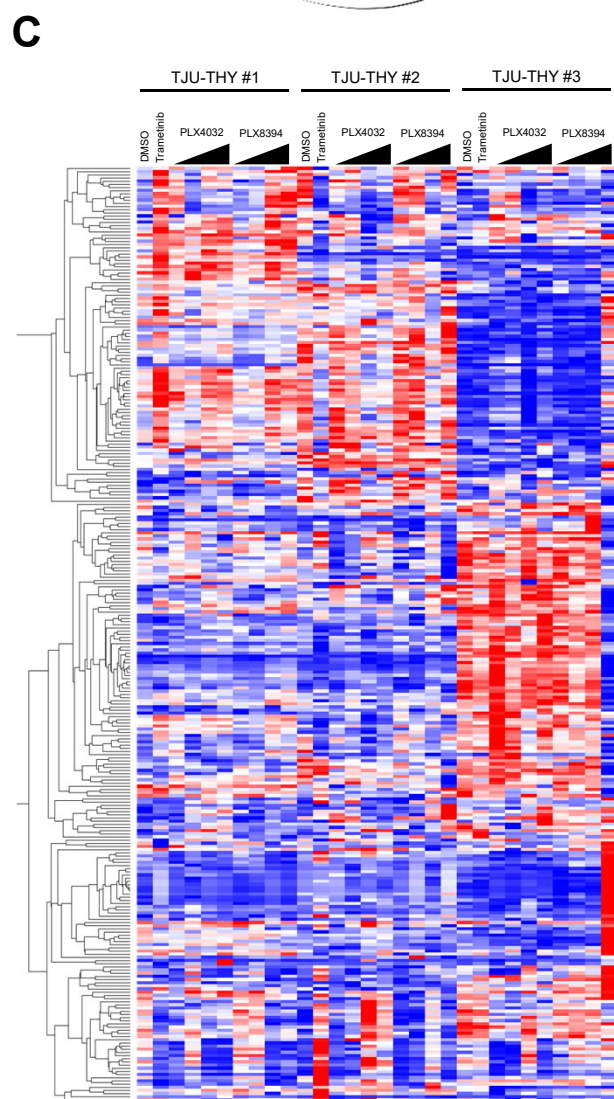

Supplement: Supplementary file 7 — Supplemental Figure 7 [file 41420_2024_1945_MOESM7_ESM.pdf]
