# Supplementary material for: Mcl-1 mediates intrinsic resistance to RAF inhibitors in mutant BRAF papillary thyroid carcinoma
Source: Cell Death Discov. 2024 Apr 15;10:175. doi: 10.1038/s41420-024-01945-0 (PMC11018618; doi:10.1038/s41420-024-01945-0)
Supplement: Supplementary file 2 — Supplemental Figure 2 [file 41420_2024_1945_MOESM2_ESM.pdf]

Cavallo Supplemental Fig.2

A

Each Antibody is Spotted in Duplicated Horizontally												
	A	B	C	D	E	F	G	H	I	J	K	L
1	POS1		POS2		POS3		ABL1		ACK		ALK-1	
2	NEG		NEG2		Axl		Blk		BMX		Btk	
3	Csk		Dtk		EGFR		EphA1		EphA2		EphA3	
4	EphA4		EphA5		EphA6		EphA7		EphA8		EphB1	
5	EphB2		EphB3		EphB4		EphB6		ErbB2		ErbB3	
6	Erb4		FAK		FER		FGFR1		FGFR2		FGFR2 (alpha isoform)	
7	Fgr		FRK		Fyn		Hck		HGFR		IGF-1 R	
8	Insulin R (CD220)		Itk		JAk1		JAK2		JAK3		LCK	
9	LTK		Lyn		MATK		M-CSF R		MUSK		NGFR (TNFRSF16)	
10	PDGFRA		PDGFRB		PYK2		RET		ROR1		ROR2	
11	ROS		RYK		SCFR (CD117)		SRMS		SYK		Tec	
12	Tie-1		Tie-2		TNK1		TRKB		TXK		NEG	
13	Tyk2		TRYO 10 (DDR2/TKT)		VEGFR		VEGFR		ZAP70		POS4	
