# Supplementary material for: Mcl-1 mediates intrinsic resistance to RAF inhibitors in mutant BRAF papillary thyroid carcinoma
Source: Cell Death Discov. 2024 Apr 15;10:175. doi: 10.1038/s41420-024-01945-0 (PMC11018618; doi:10.1038/s41420-024-01945-0)
Supplement: Supplementary file 8 — Supplemental Figure Legends [file 41420_2024_1945_MOESM8_ESM.pdf]

SUPPLEMENTAL FIGURES

Supplemental Figure 1: BRAFi elicits heterogenous ERK1/2 pathway suppression in human PTC cell lines. (A) PTC cell lines treated with DMSO or increasing concentrations of trametinib (5, 10, 25, 50nM), PLX4720 (100, 500, 1000, 5000nM), or PLX8394 (100, 500, 1000, 5000nM) for 48 hours prior to preparation for western blot analysis. Representative western blot images are shown. Densitometry was used to quantify the ratio of pERK1/2 to ERK2 at each treatment condition and is quantified (right). Data points are presented as average fold change compared to DMSO condition and are an average of three independent experiments. Error bars are ±SEM. trametinib – black circles, PLX4720 – gray circles, and PLX8394 – white circles.

Supplemental Figure 2: Resistant MDA-T32 PTC cell line demonstrates innate resistance. (A) Antibody legend of RayBiotech Inc. phosphorylation array.

Supplemental Figure 3: VCAM-1 is not associated with BRAFi resistance. (A) The most enriched genes generated by GSEA analysis (Figure 2C) are shown. Genes are further annotated with green (1 hit) or purple (2 hits) to indicate how often each gene contributed to pathway enrichment depicted in Figure 2C. **(B)** Western blot analysis of PTC cell lines treated with PLX4720 for 24 hours show upregulated VCAM expression exclusively in MDA-T32 cells. **(C)** Western blot analysis represents successful siRNA knock down of VCAM-1 in MDA-T32 cells. **(D)** Crystal violet staining of MDA-T32 and MDA-T41 cells to assay proliferation under drug treatment after siRNA knockdown is represented by fold plate coverage. **(E)** To monitor cell cycle progression, S-phase entry analysis of MDA-T32 and MDA-T41 cells after siRNA knockdown was performed and is represented by fold EdU incorporation. **(F)** MDA-T41 cells were engineered to inducibly overexpress VCAM-1 under treatment with doxycycline (dox).

Western blot analysis demonstrated increased expression of VCAM-1 in MDA-T41 cells under treatment with dox. **(G)** S-phase entry analysis was performed on MDA-T41 cells overexpressing VCAM-1 under treatment with monomer-selective PLX4720 or MEK inhibitor trametinib. S-phase entry is represented by fold EdU incorporation. The error bars are ±SEM (B,C,E). The * is indicative of $p<0.05$, ** of $p<0.01$, *** of $p<0.001$ and # $p<0.0001$ as determined by one-way ANOVA analysis with multiple comparisons (D,E, and G).

Supplemental Figure 4: Effects of AZD5991 on MDA-T32 and MDA-T41. (A) Western blot analysis of "minority MOMP"-associated proteins in PTC cell lines after a 30 minute with DMSO or AZD5991 (1nM,10 nM,100nM, 1000nM, and 2000nM) **(B)** Similar to (A) except 24-hour incubation with DMSO or AZD5991 (1000nM, 2000nM, or 5000nM).

Supplemental Figure 5: Lysosomal susceptibility to Mcl-1 inhibitor indicates MOMP-like phenotype. **(A)** Western blot analysis of LAMP-1 and pRb in PTC cell lines after 24-hour treatment with DMSO or PLX4720 (1000nm). **(B, C)** Flow cytometry analysis (B) and representative image (C) of Lysosensor green staining after 24-hour treatment with DMSO or AZD5991 (2000nm or 5000nm). Data points are representative of the geometric mean of Lysosensor green staining, error bars are ±SEM, n=2. The * is indicative of $p<0.05$, ** of $p<0.01$, *** of $p<0.001$ and # $p<0.0001$ as determined by t-test.

Supplemental Figure 6: Sublethal treatment with navitoclax does not provide resistance to BRAFi. (A) Quantified flow cytometry analysis of 7-aad staining of parental MDA-T41 and habitual OPN/IFNγ or navitoclax treated variants. Data points are representative of percentage healthy cells (7-aad$^-$) from three independent experiments with error bars signifying ±SEM, n=3. **(B)** Quantification of crystal violet staining of parental MDA-T41 cells versus MDA-T41 cells chronically exposed to navitoclax after 6 days treated with increasing doses of PLX4720

(100nM, 250nM, 500nM, and 1000nM), represented by fold plate coverage compared to DMSO treatment. Error bars are -/+ SEM, and two-way ANOVA analysis with multiple comparisons did not identify significant differences.

Supplemental Figure 7: *Ex vivo* analysis of PTC patient samples reveals heterogenous response to BRAFi. (A) Schematic representation of tissue extracted from PTC patients are harvested and processed for ex vivo sequencing. Ex vivo PTC tumor samples are treated with various BRAFi to compare efficacy. **(B)** Sanger sequencing of three different tissue extractions from PTC patients (TJU-THY#1, TJU-THY#2, TJU-THY#3) reveal heterozygous $BRAF^{V600E}$ mutations in patient samples #1 and #2, and an $NRAS^{Q61R}$ mutation in patient #3. **(C)** Tumor samples were used for RPPA analysis. Protein signals were sorted by hierarchical clustering and represented via heatmap to highlight differentially expressed proteins was created using Broad Institute Morpheus software. Three patient samples are displayed TJU-THY #1, #2, and #3, proteins were sorted by hierarchical clustering.
