# Supplementary material for: Mcl-1 mediates intrinsic resistance to RAF inhibitors in mutant BRAF papillary thyroid carcinoma
Source: Cell Death Discov. 2024 Apr 15;10:175. doi: 10.1038/s41420-024-01945-0 (PMC11018618; doi:10.1038/s41420-024-01945-0)
Supplement: Supplementary file 9 — Original Data Files [file 41420_2024_1945_MOESM9_ESM.pdf]

Figure 1D

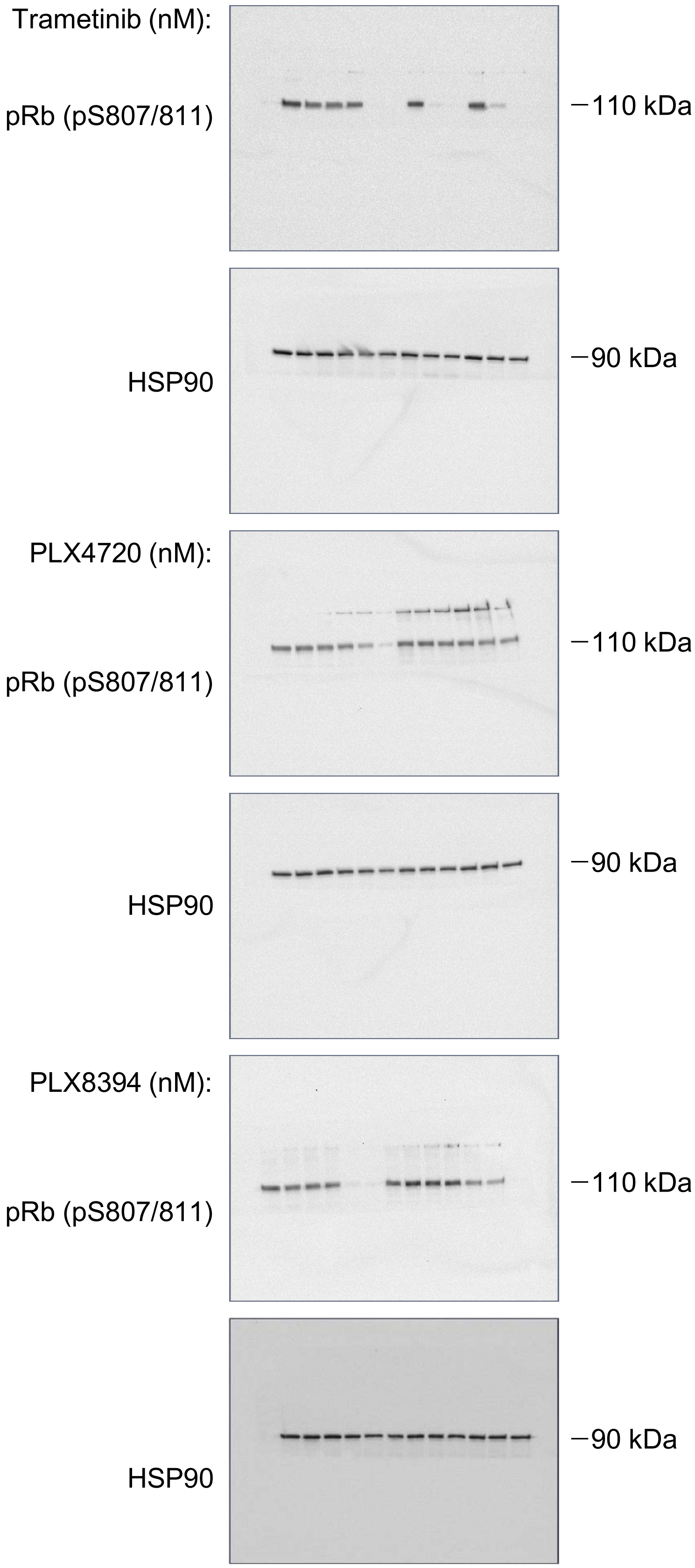

Trametinib (nM):
pRb (pS807/811)
110 kDa
HSP90
90 kDa
PLX4720 (nM):
pRb (pS807/811)
110 kDa
HSP90
90 kDa
PLX8394 (nM):
pRb (pS807/811)
110 kDa
HSP90
90 kDa

Figure 2A

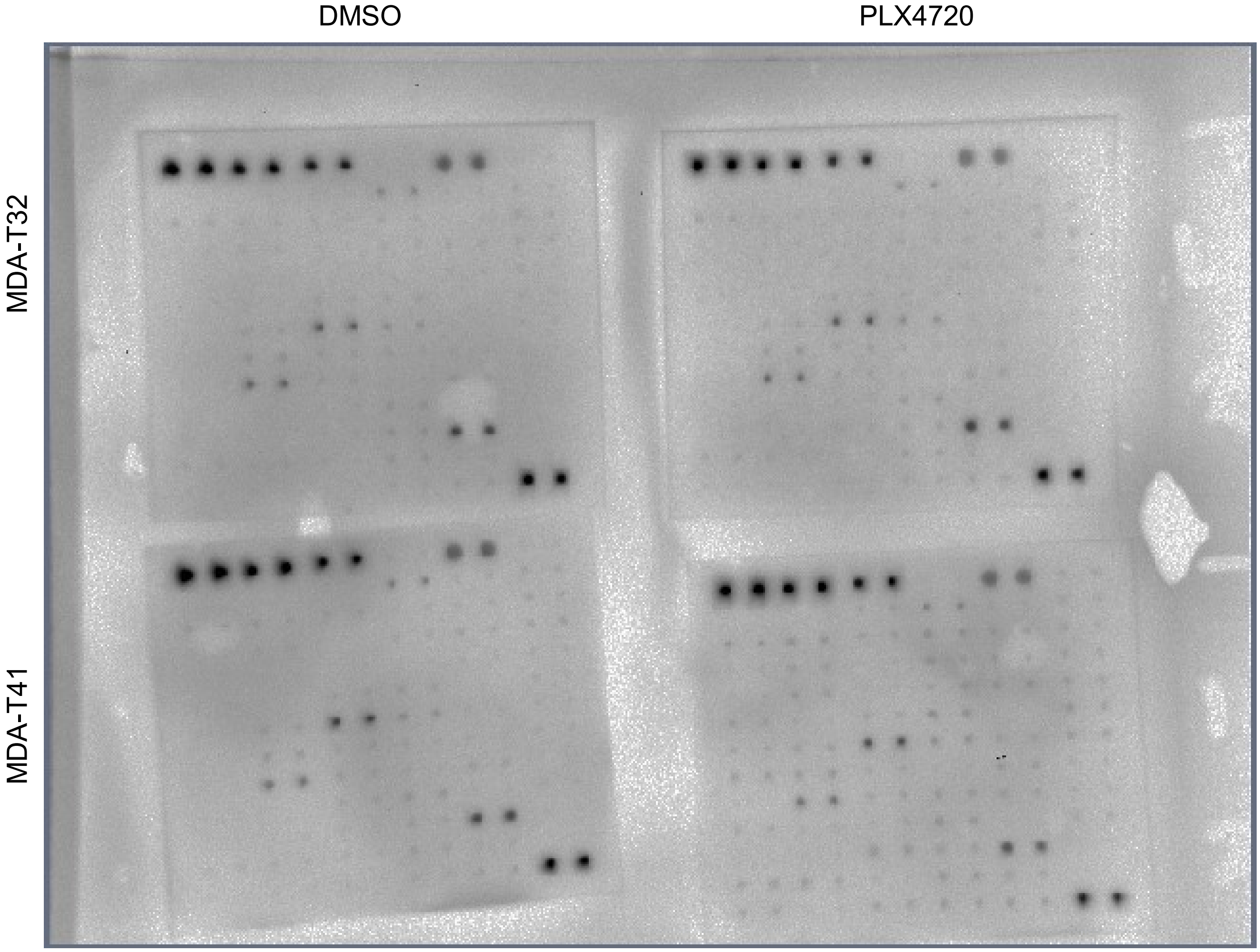

Figure 3A

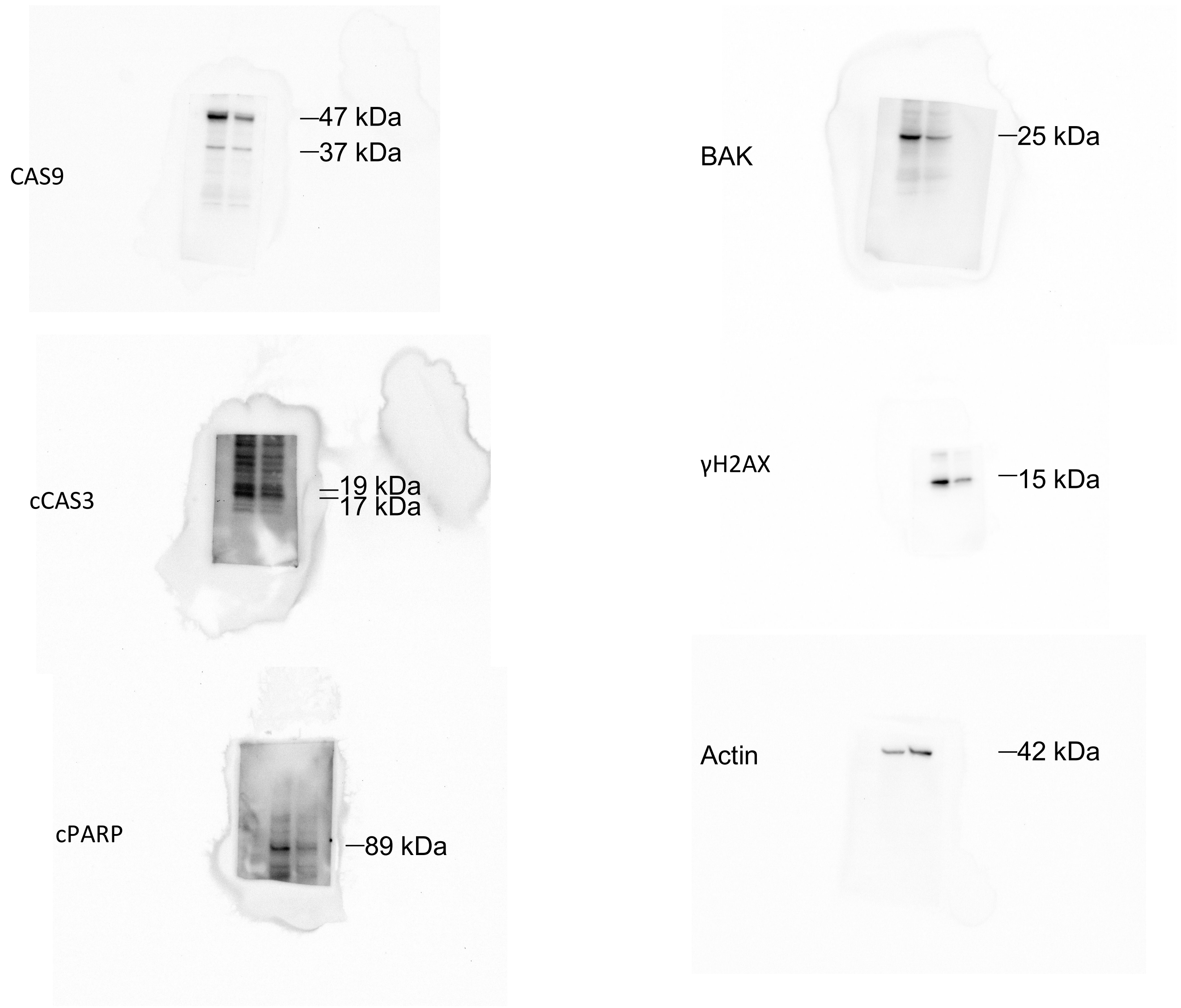
CAS9
47 kDa
37 kDa
BAK
25 kDa
cCAS3
19 kDa
17 kDa
γH2AX
15 kDa
cPARP
89 kDa
Actin
42 kDa

Figure 3D

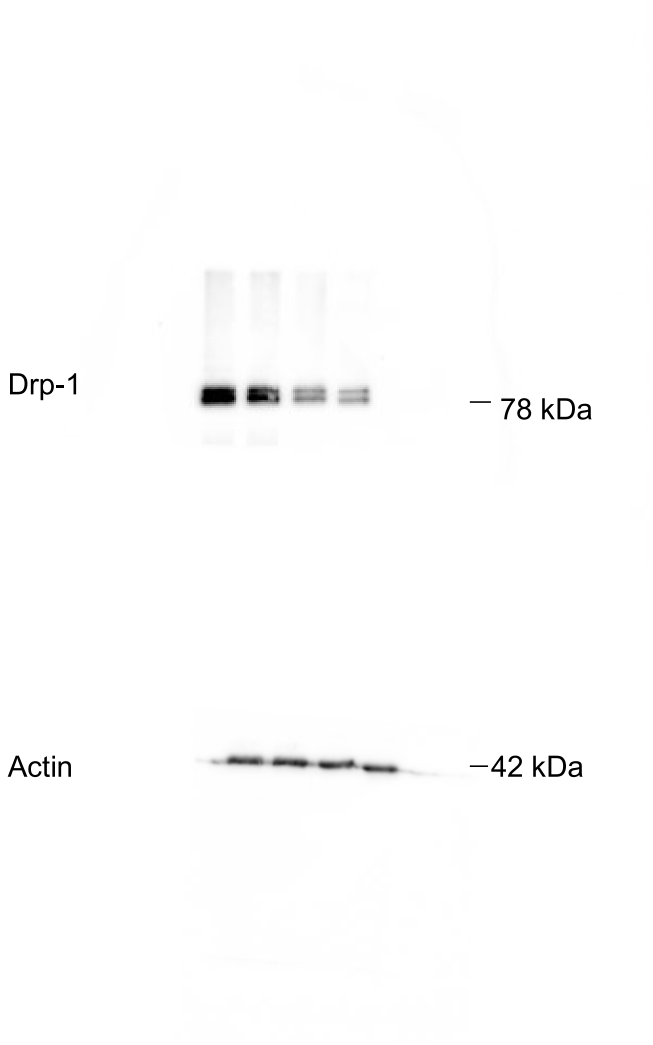

Figure 4A

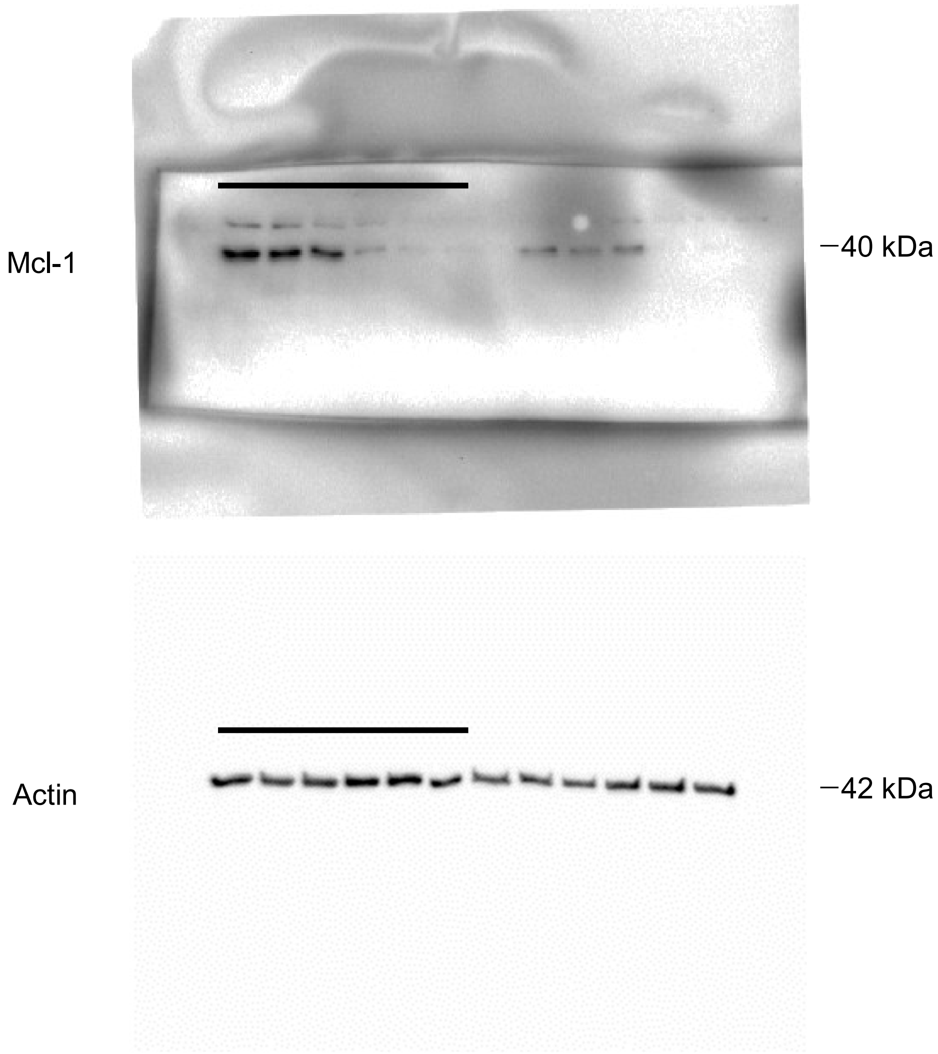

Note: lanes 1-6 are used in Fig. 4A

Figure 4D

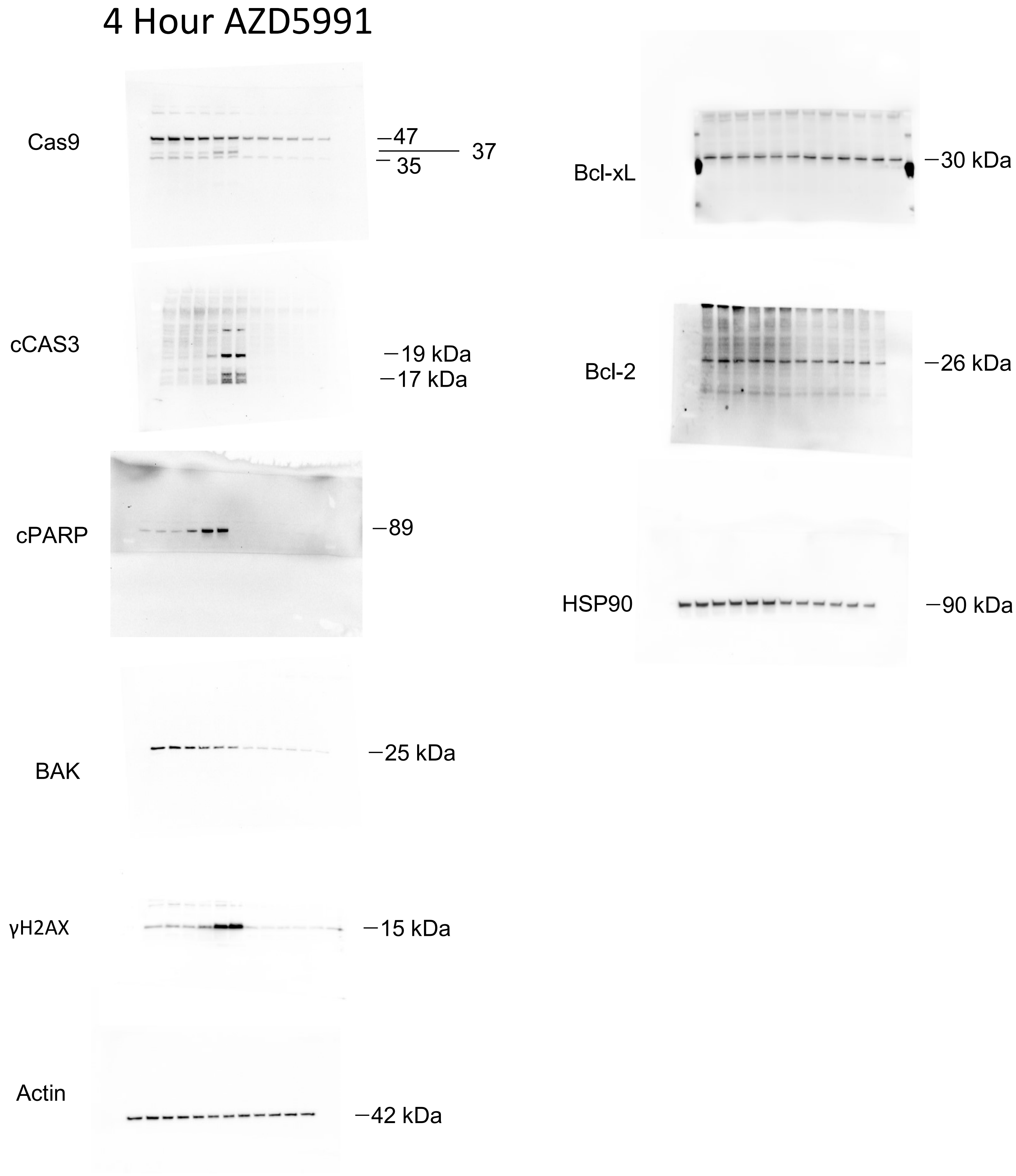
4 Hour AZD5991
Cas9
47
37
35
cCAS3
19 kDa
17 kDa
cPARP
89
BAK
25 kDa
γH2AX
15 kDa
Actin
42 kDa
Bcl-xL
30 kDa
Bcl-2
26 kDa
HSP90
90 kDa

Figure 4E

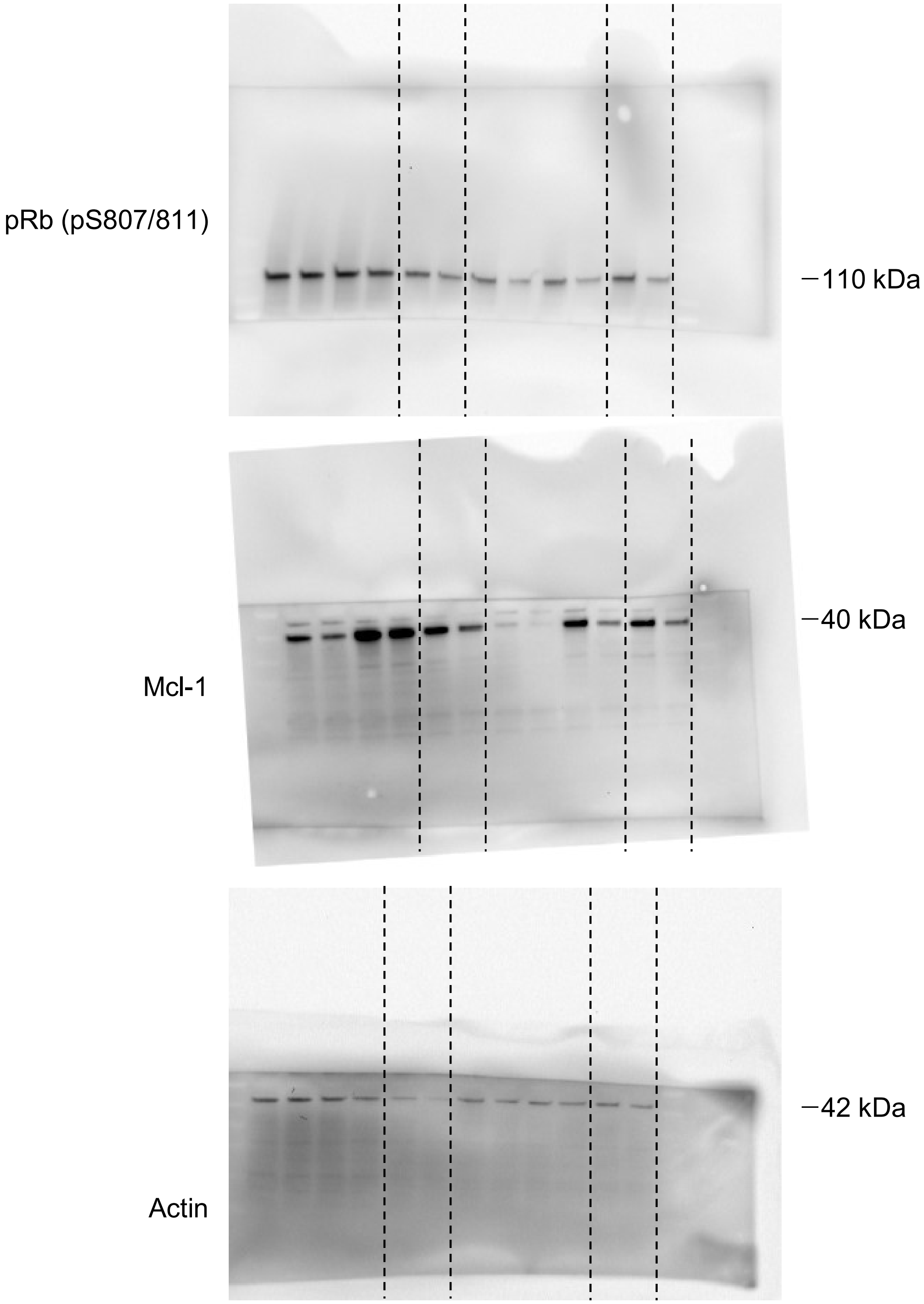

Note: lanes 5 and 6 are cropped in Fig. 4E (indicated with Black vertical line). Lanes 11-12 unused

Figure 4F

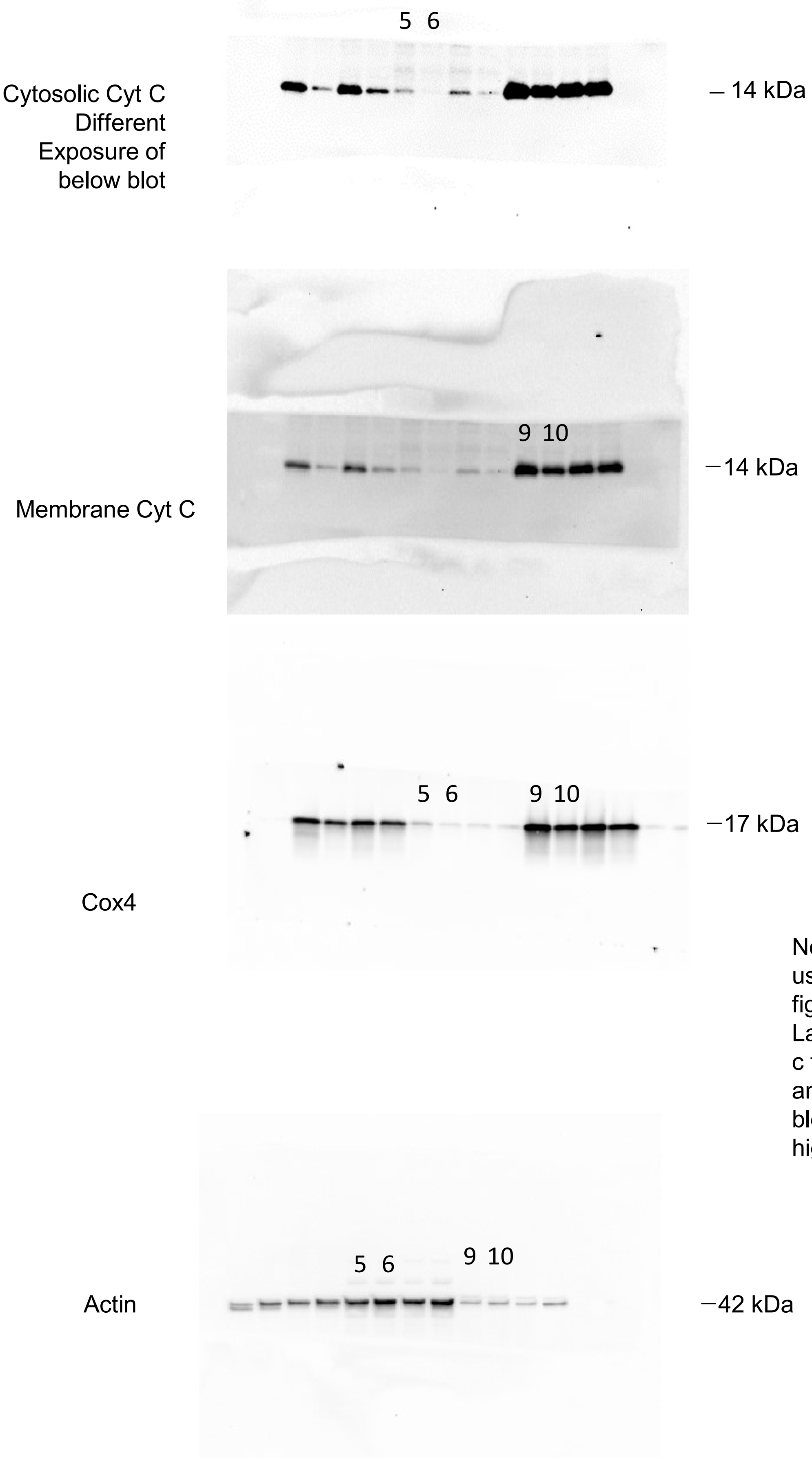

Note: Lanes 5,6, 9, and 10 were used in all blots represented in figure 4F for Cox4 and actin. Lanes 9 and 10 were used for Cyt c for membrane fraction. Lanes 5 and 6 were used in the topmost blot under a different exposure to highlight cystolic cytochrome c.

Figure 5C

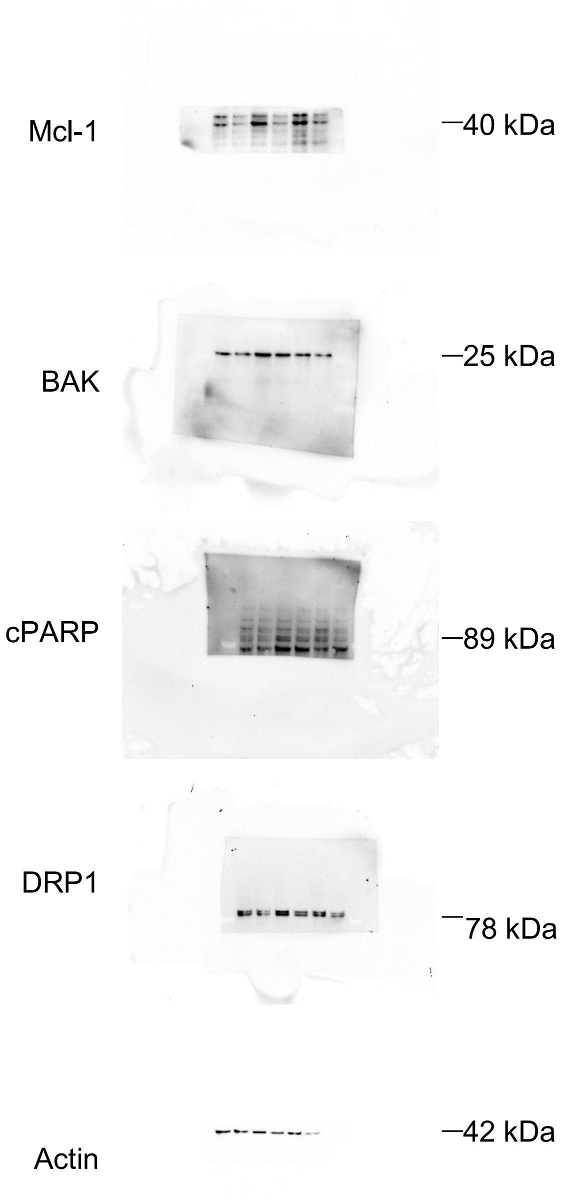

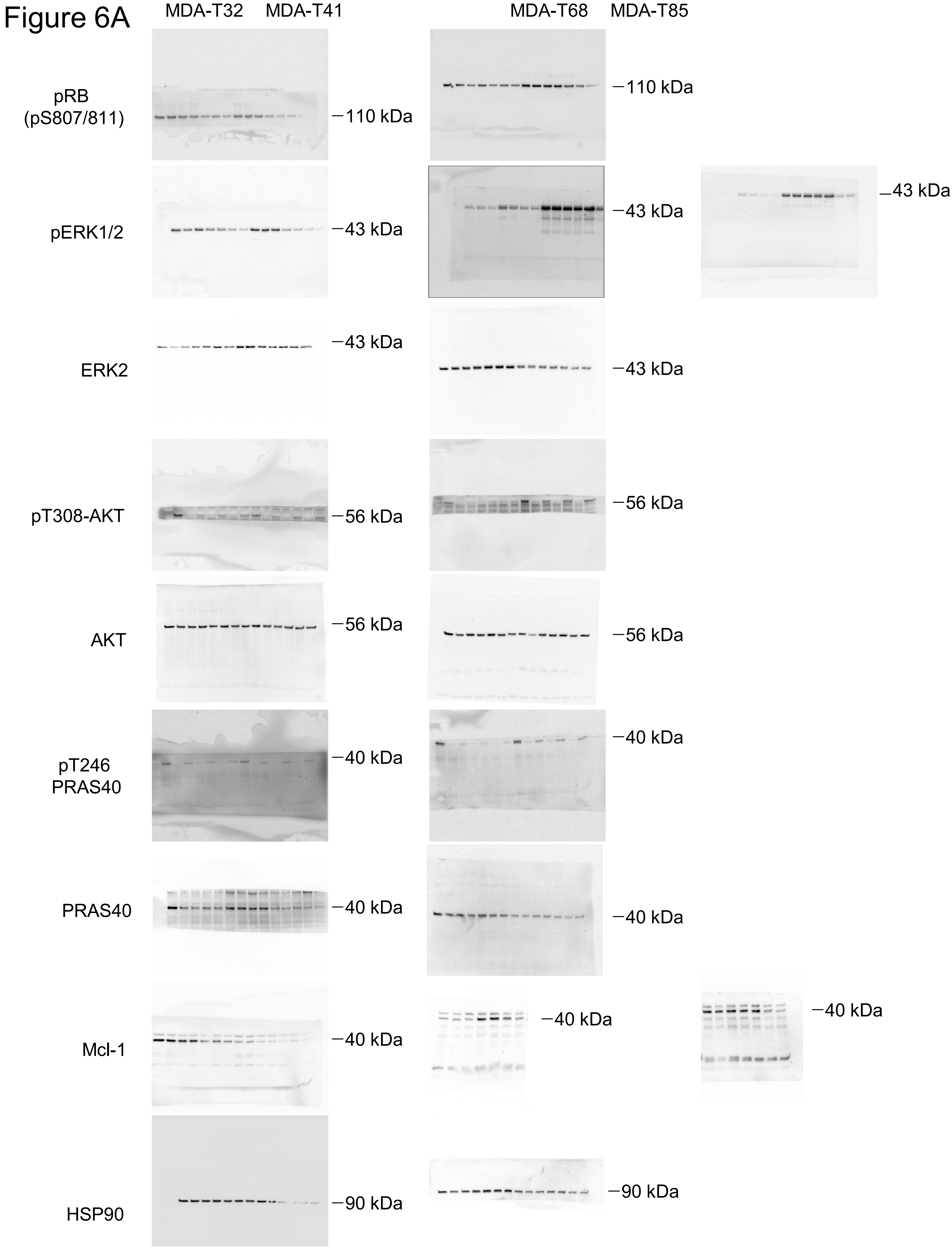
Figure 6A
MDA-T32
MDA-T41
MDA-T68
MDA-T85
pRB (pS807/811)
110 kDa
pERK1/2
43 kDa
ERK2
pT308-AKT
56 kDa
AKT
pT246 PRAS40
40 kDa
PRAS40
Mcl-1
HSP90
90 kDa

Figure 7A

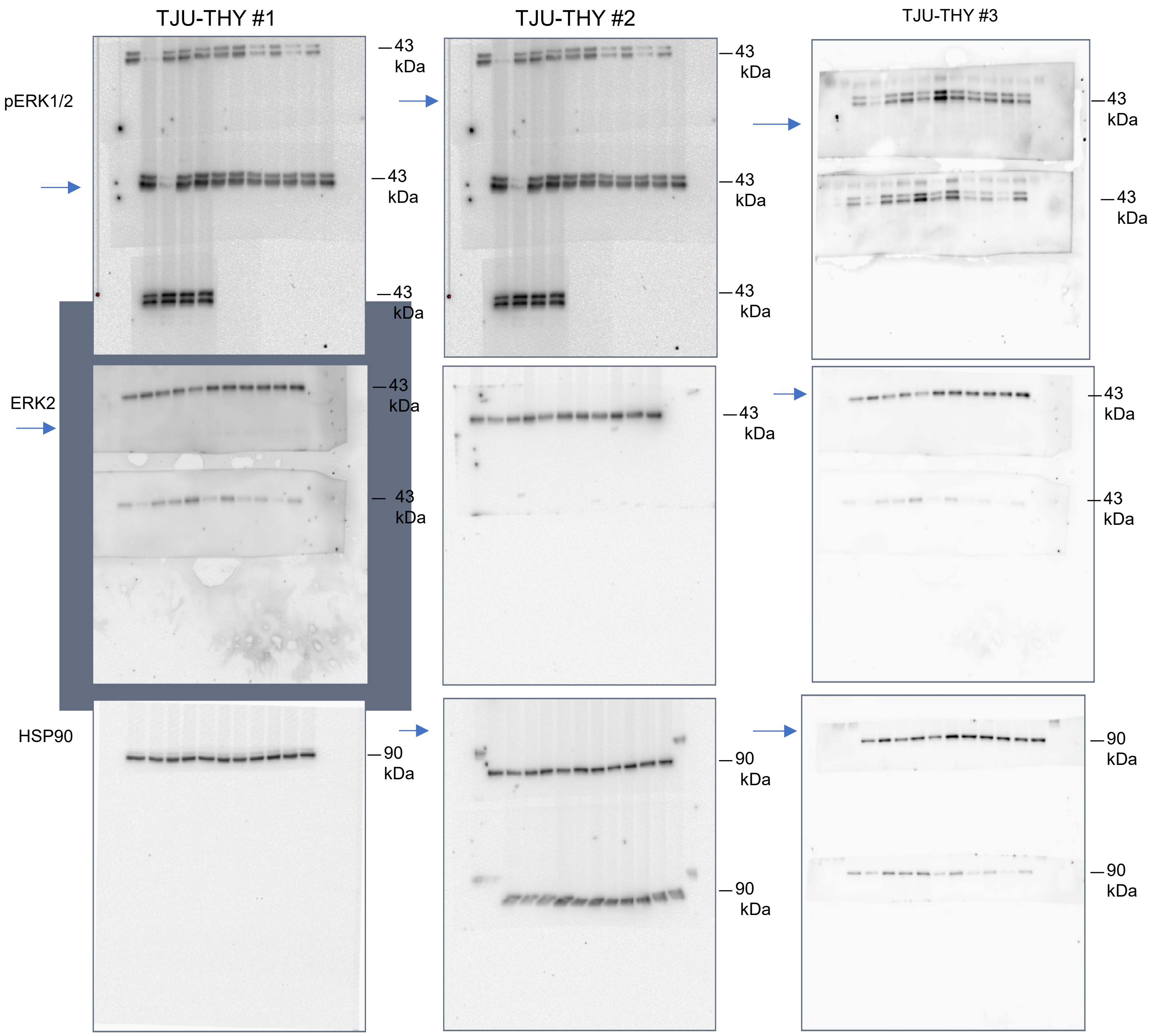

Figure 7C

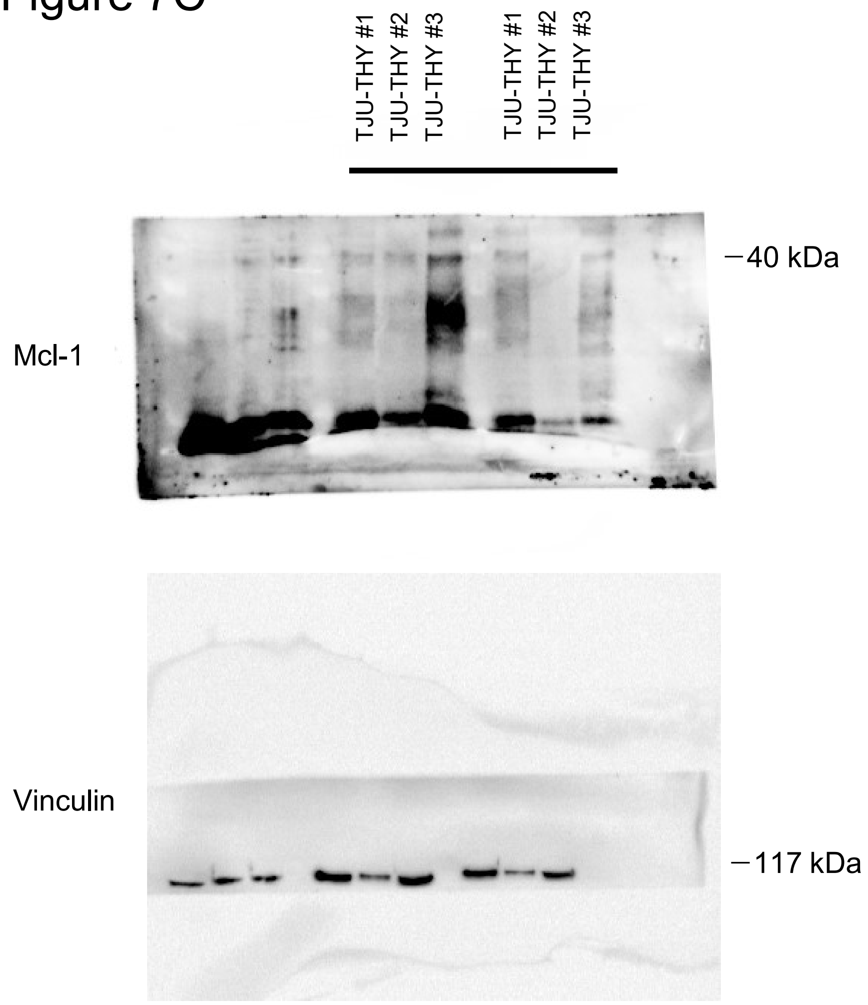
TJU-THY #1
TJU-THY #2
TJU-THY #3
TJU-THY #1
TJU-THY #2
TJU-THY #3
Mcl-1
40 kDa
Vinculin
117 kDa

Supplemental Figure 1A

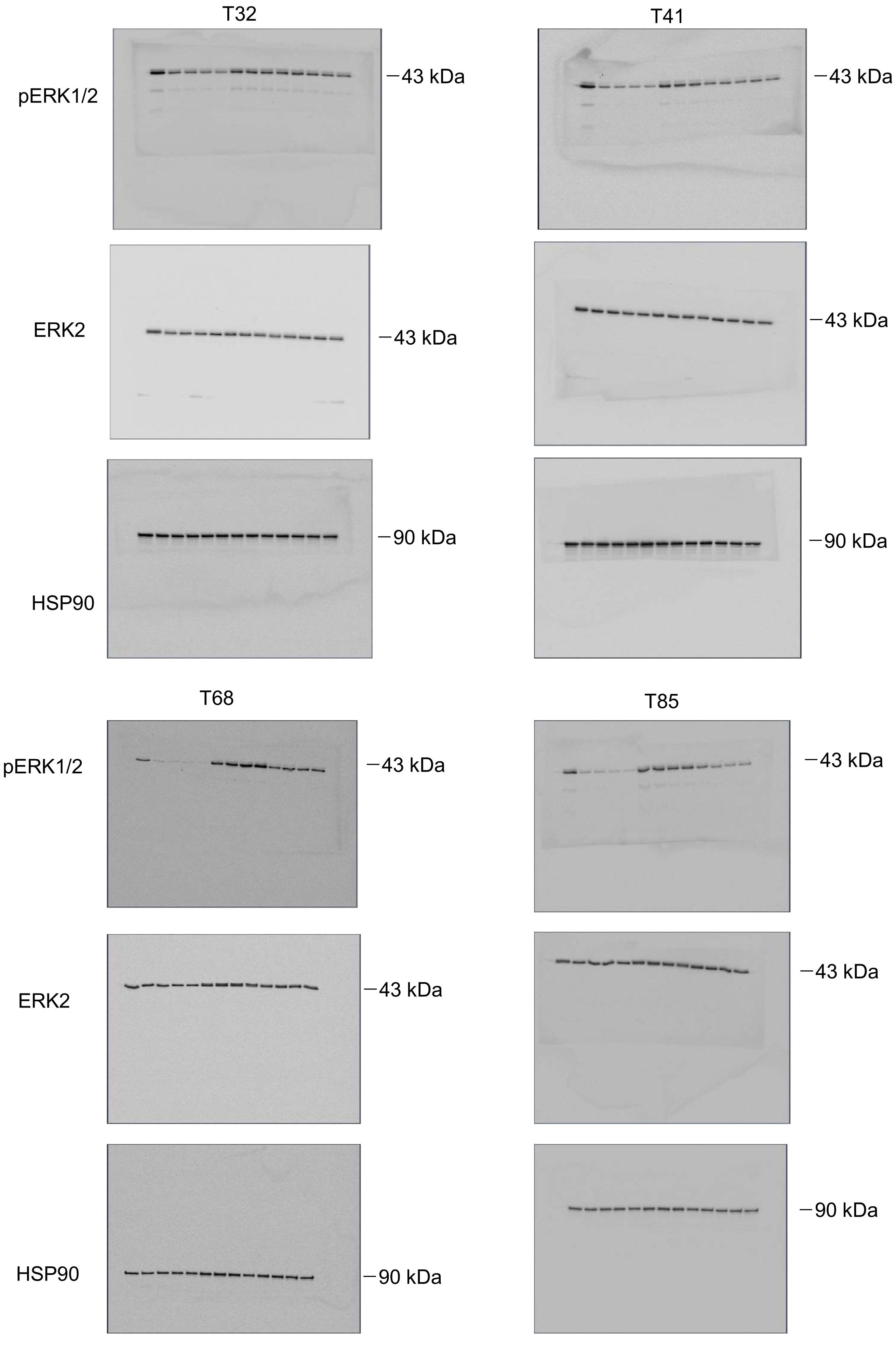

Supplemental Figure 3B

VCAM1

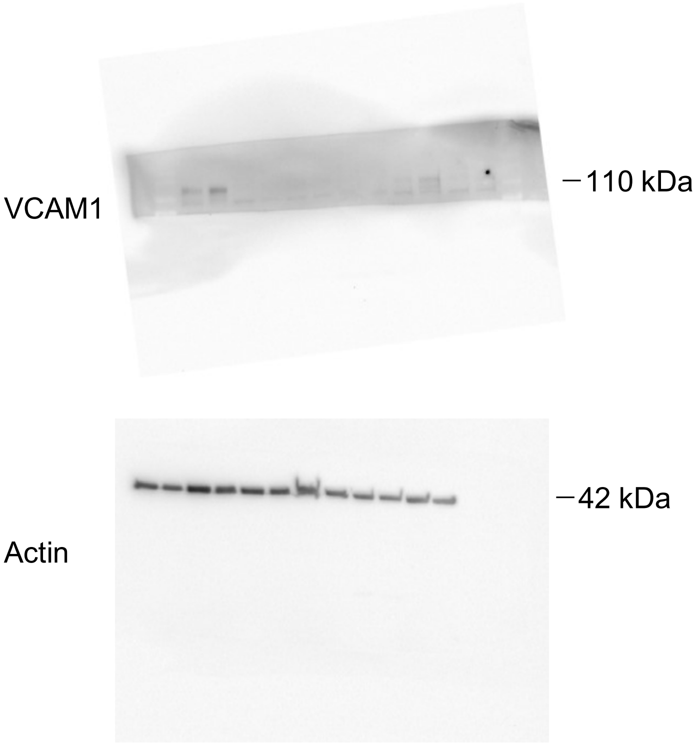

—110 kDa

Actin

—42 kDa

Supplemental Figure 3C

VCAM1

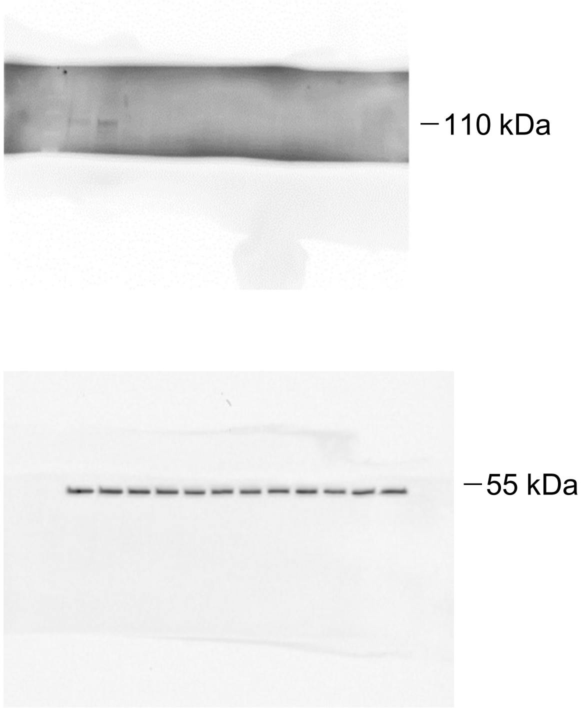

—110 kDa

Tubulin

—55 kDa

Supplemental Figure 3F

VCAM1

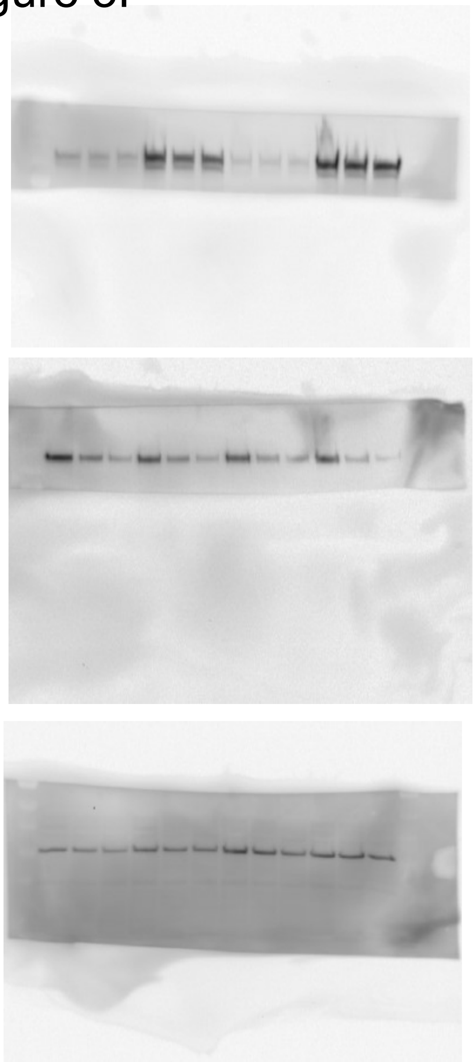

—110 kDa

pRb (pS807/811)

—110 kDa

GAPDH

—36 kDa

Supplemental Figure 4

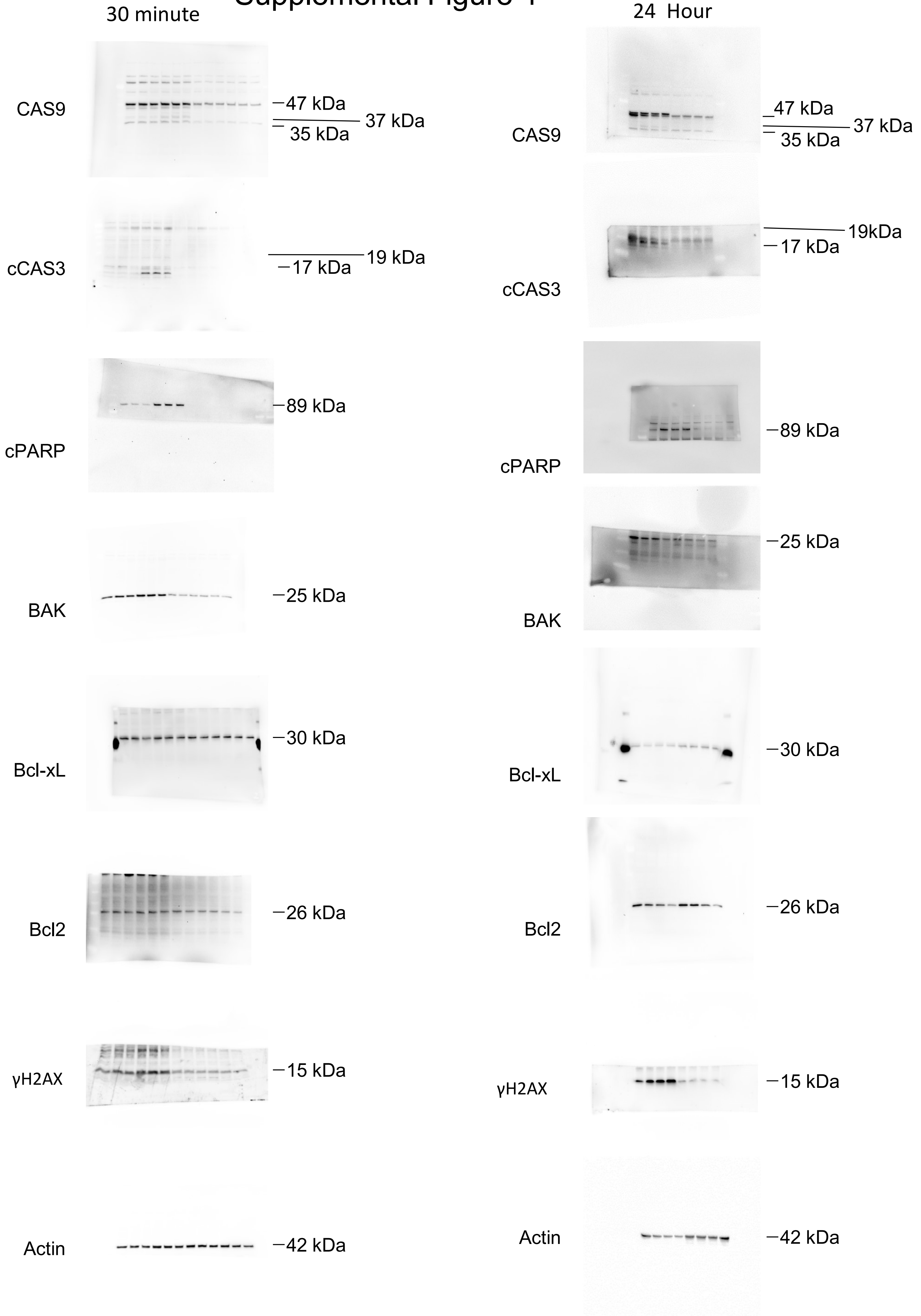

Supplemental Figure 5A

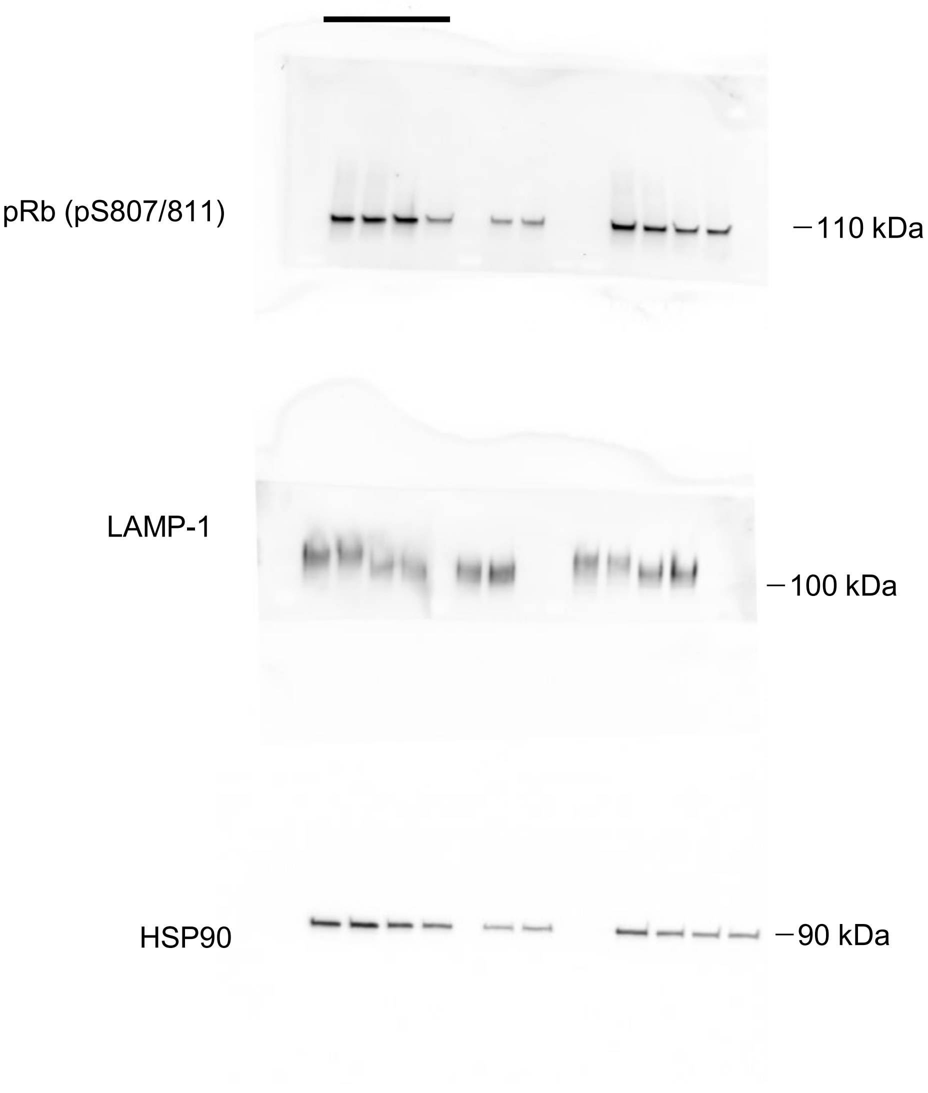

Note: lanes 1-4 are used in Supplemental Fig. 5A
